# Supplementary material for: Hidden heterogeneity and circadian-controlled cell fate inferred from single cell lineages
Source: Nat Commun. 2018 Dec 18;9:5372. doi: 10.1038/s41467-018-07788-5 (PMC6299096; doi:10.1038/s41467-018-07788-5)
Supplement: Supplementary file 1 — Supplementary Information [file 41467_2018_7788_MOESM1_ESM.pdf]

Supplementary Information
"Hidden heterogeneity and circadian-controlled cell fate inferred from single cell lineages"

Shaon Chakrabarti[1*], Andrew L. Paek[2,3*], Jose Reyes[3], Kathleen A. Lasick[2]
Galit Lahav[3,4+], and Franziska Michor[1,4,5+]

[1]Department of Biostatistics and Computational Biology, Dana-Farber Cancer Institute, Department of Biostatistics, Harvard T. H. Chan School of Public Health, Boston, MA, USA, and Department of Stem Cell and Regenerative Biology, Harvard University, Cambridge, MA, USA. [2]Current address: University of Arizona, Tucson, AZ, USA. [3]Department of Systems Biology, Harvard Medical School, Boston, MA, USA. [4]Broad Institute of Harvard and MIT, Cambridge, MA, USA, and Ludwig Center at Harvard, Boston, MA, USA. [5]Center for Cancer Evolution, Dana-Farber Cancer Institute, Boston, MA, USA. [*]Equal contribution. [+]Authors for correspondence. Email Galit_Lahav@hms.harvard.edu, michor@jimmy.harvard.edu.

Contents

1. Calculation of lineage correlations in times to fate 2
2. Cell fate is correlated in related cells 3
3. Cell-cycle stage does not affect p53 dynamics or cell death in response to cisplatin 3
4. Comparison of model fits to IMT and AT distribution data 4
5. Statistical algorithm to infer unbiased IMT and AT distributions 5
    1. Basic introduction to survival and competing risks analysis
    2. Basic introduction to copulas
    3. Algorithm based on competing risks analysis and copulas to infer unbiased IMT and AT distributions
    4. Modeling probability of surviving treatment
    5. Modeling delayed response of cells after drug administration
    6. Accounting for sister correlations improves parameter estimation of IMT distributions

7. Results from the statistical algorithm — maximum likelihood and MCMC simulations

6. Age-dependent birth-death process model 15

1. Basic model with no circadian coupling

2. Modeling cell fate dependence on stochastic protein production/degradation

3. Modeling circadian gating of the cell cycle.

4. Varying the periods of the oscillator gating of the cell cycle

5. Gating of cell apoptosis by the circadian clock.

6. Random phase change of the circadian clock during cell division

7. Random circadian phases for different cellular lineages

8. Modeling correlated cell fates of sisters

7. Supplemental experimental methods 21

1. Calculation of lineage correlations in times to fate

To calculate lineage correlations in intermitotic and apoptosis times (IMT and AT, respectively) from the single cell lineage tracing data, all mother-daughter, sister-sister and cousin-cousin pairs were first identified, allowing for the possibility that unrelated cells from different clones may be born in the same time frame. Following previous work[1], unique pairs were then identified for each relationship such that no cell was counted twice in the calculation of the correlation coefficients. Finally, statistical significance of the correlations and 95% confidence intervals were computed using the "cor.test" function in R, which uses the t-test to calculate statistical significance. Note that a set of unique pairs can be found in many different ways depending on the particular choice of cells for defining mother-daughter or cousin pairs; the correlations were similar within the 95% confidence intervals for any of the choices. The correlations among the lineages before and after drug treatment are shown in Fig. 1. For cells that straddle the cisplatin dosing event, sisters have an IMT correlation $\rho \sim 0.66$ from 52 pairs, P-val = 5.7×10^{-8}, 95% CI [0.44, 0.79] as shown in Supplementary Figure 1a. Correlation in IMT of cousins among straddling cells is $\rho \sim 0.28$ from 37 pairs, P-val = 0.03, 95% CI [0.08, 0.5], as shown in Supplementary Figure 1b.

2. Cell fate is correlated in related cells

To test the impact of cell state prior to cisplatin treatment on the response of HCT116 cells to cisplatin, we calculated how frequently related cells shared the same fate by lineage relationship and compared these frequencies to unrelated cells. We first considered only death or survival as a cell fate (Supplementary Figure 2a). Since 62.5% of cells died in response to cisplatin treatment, then 53% of cells should share the same fate if the response of each cell was an independent event as the probability of both cells dying is ~39% (0.625 * 0.625) and the probability of both cells surviving is ~14% (0.375 * 0.375). Indeed, unrelated cells shared the same fate 52% of the time (N = 12,495 cell pairs). In contrast, sister cells shared the same fate over 80% of the time and the correlation in cell fate decayed with lineage distance. Cells separated by 4 divisions, or 3rd cousins, shared the same fate in similar proportions to unrelated cells (Supplementary Figure 2a).

We next included cell division as one of the outcomes following cisplatin treatment. Following cisplatin treatment cells can divide, die, do neither or do both (Supplementary Figure 2b). Again, we found that related cells share the same fate much more frequently than expected for independent events and the correlation in cell fate decayed with lineage distance (Supplementary Figure 2c).

3. Cell cycle stage does not affect p53 dynamics or cell death in response to cisplatin

The efficacy of many chemotherapy drugs varies with cell cycle stage[2]. To determine if the rate of p53 accumulation and cell fate is linked to cell cycle stage, we engineered a system to measure cell cycle stage, p53 dynamics, and cell fate in live cells. We used HCT116 p53-VKI human colon cancer cells, a previously established clonal cell line in which one allele of *TP53* is tagged with the Venus fluorescent protein[3]. To track cell cycle phase, we expressed Cerulean fused with the N-terminal domain of human geminin (Cer-hGem). Cer-hGem is degraded by the anaphase-promoting complex (APC) during M-phase and G1 when APC activity is high. APC inactivation upon S-Phase entry triggers accumulation of Cer-hGem which remains high until the next M-phase(Supplementary Figure 3a)[4]. The Cer-hGem reporter had no measurable effect on cell cycle length (Supplementary Figure 3b).

We imaged untreated cells for 24 hours to identify their cell cycle stage. We then treated cells with cisplatin and imaged an additional 72 hours (Supplementary Figure 3c) as most cell death occurs within this time[3]. Cell cycle stage was slightly synchronized at the initial stages of the experiment (Supplementary Figure 3c), likely due to the synchronizing effects of media changes and cell plating. After cisplatin treatment, cells slowed their rate of progression through the cell cycle, consistent with cell cycle arrest. A subset of cells degraded Cer-hGem without undergoing mitosis (Mitosis Skip, Supplementary Figure 3c) as shown previously[5]. Approximately 50% of cells underwent apoptosis (Supplementary Figure 3c).

We compared p53 dynamics and cell fate between cells that were in G1 or S/G2 at the time of cisplatin treatment. We found no connection between cell cycle stage at the time of treatment and either cell death or onset of p53 accumulation (Supplementary Figure 3d,e). It is possible that cisplatin acts slowly to damage DNA and that this delay masks our ability to observe a

connection between cell cycle stage and cell fate. However, the cell cycle stage of cells at later time points also had no effect on cell death or p53 onset (Supplementary Figure 3f,h). These data suggest that the variation observed in p53 dynamics and cell death in response to cisplatin is not due to variation in cell cycle stage at the time of or during treatment. In contrast, we did observe a correlation between the cell cycle stage at the time of cisplatin addition and the probability that a cell will divide after treatment (Supplementary Figure 3j). For example, although only 34% of cells were in G1 when cisplatin was added, these G1 cells accounted for 83% of the cells that did not die or divide following cisplatin treatment (Supplementary Figure 3j).

4. Comparison of model fits to IMT and AT distribution data

The most common two and three parameter functions that have previously been used to describe cell division and apoptosis time distributions — Exponentially Modified Gaussian (EMG), gamma, and shifted gamma – were tested against our single cell data. The functional forms of the probability density functions (pdf) are given by

EMG: $f(t;\mu,\sigma,\lambda)=\frac{\lambda}{2}\,e^{\frac{\lambda}{2}(-2t+2\mu+\lambda\sigma^2)}Erfc\left(\frac{-t+\mu+\lambda\sigma^2}{\sigma\sqrt{2}}\right)$,

where *Erfc* is the complementary error function. The EMG is a convolution of a Gaussian density with mean μ and variance σ^2, and an exponential with parameter λ. The mean of the EMG is given by $\mu+1/\lambda$ and the variance is σ^2+1/λ^2.

Gamma: $f(t;b,g)=\frac{\left(\frac{t}{b}\right)^{g-1}e^{\left(-\frac{t}{b}\right)}}{b\,\Gamma(g)}$,

where Γ is the gamma function. The mean of the gamma distribution is given by bg and the variance by gb^2.

Shifted gamma: $f(t;b,g,u)=\frac{\left(\frac{t-u}{b}\right)^{g-1}e^{\left(-\frac{t-u}{b}\right)}}{b\,\Gamma(g)}$,

where Γ is the gamma function. The mean of the shifted gamma distribution is $bg+u$ and the variance is gb^2.

All nonlinear model fitting and model selection calculations (using the Akaike Information Criterion, AIC[6]) were performed using the "NonlinearModelFit" function in Mathematica. The Exponentially Modified Gaussian (EMG) function describes the data before cisplatin dosing significantly better than any other model (Supplementary Table 1). The best fitting EMG parameters are $\mu=28.57$, $\sigma=2.45$, $\lambda=0.27$. The closest competitor, the Gamma function, has an AIC larger than the AIC for EMG by 6.5, implying that the Gamma function is only $e^{-6.5/2}=0.038$ times (3.8%) as likely to best explain the data as the EMG function[6].

Supplementary Table 1: Comparison of model fits to experimental data of the inter-mitotic time (IMT) distribution before cisplatin treatment. EMG is the Exponentially Modified Gaussian function.

Function	Number of parameters	AIC	BIC
**EMG**	3	-66.79	-66.47
**Gamma**	2	-60.29	-60.05
**Shifted gamma**	3	-56.13	-55.80

Similarly, the EMG function describes the IMT distribution of cells that straddle the cisplatin dosing event significantly better than the other functions (Supplementary Table 2). The best fitting EMG parameters for the straddling cells are $\mu = 29.26,\ \sigma = 3.5,\ \lambda = 0.093$. The next best model was the shifted gamma, which was only 6 % as likely to best explain the IMT data as the EMG function.

Supplementary Table 2: Comparison of model fits to experimental data on the inter-mitotic time (IMT) distribution of cells that straddle the cisplatin dosing event.

Function	Number of parameters	AIC	BIC
**EMG**	3	-74.91	-74.59
**Shifted gamma**	3	-69.28	-68.96
**Gamma**	2	-60.17	-59.90

Finally, the same analysis showed that the EMG also best describes the time to death distribution data of cells existing purely after cisplatin treatment (Supplementary Table 3). The best fitting EMG parameters for this case are $\mu = 45.836, \sigma = 27.9,\ \lambda = 0.209$. The next best model was the Gamma, which was only 8.9% as likely to best explain the time to death data as the EMG function.

Supplementary Table 3: Comparison of model fits to experimental data on the time to death distribution after cisplatin administration.

Function	Number of parameters	AIC	BIC
**EMG**	3	-66.82	-67.03
**Gamma**	2	-62.56	-62.72
**Shifted gamma**	3	-59.94	-60.16

5. Statistical algorithm to infer unbiased IMT and AT distributions

Basic introduction to survival and competing risks analysis

Survival analysis is a statistical technique for analyzing time to event data, and competing risks occur when there are multiple events that are mutually exclusive—the occurrence of one event precludes the occurrence of any of the other events. This technique is usually framed in terms of hazard functions, which describe the instantaneous rates of occurrence of various events and are central to the development of our statistical algorithm.

Let T be a non-negative random variable denoting the time until occurrence of an event, and t be an instantiation of T. Let the probability density function (pdf) of T be $f(t)$ and the cumulative distribution function (cdf) be $F(t)$, such that

$$F(t) = \Pr(T \leq t) = \int_0^t f(s)ds. \tag{1}$$

The survival function $S(t)$ is the complement of the cdf, and denotes the probability that the event has not occurred by time t:

$$S(t) = \Pr(T > t) = 1 - F(t). \tag{2}$$

The hazard function for the event is a measure of the risk that the event will occur at any point in time, given the event has not happened up to that time. Formally, the hazard function is defined as

$$h(t) = \lim_{\delta t \to 0} \frac{\Pr(t < T \leq t + \delta t \mid T > t)}{\delta t}. \tag{3}$$

The expression in Supplementary Equation (3) can easily be shown to simplify to

$$h(t) = \frac{f(t)}{S(t)}. \tag{4}$$

The hazard function is therefore simply a ratio of the pdf and survival functions, as given in Supplementary Equation (4). It can be interpreted as a rate of occurrence of the event. For a Poisson process with rate λ and an exponentially distributed waiting time distribution given by $f(t) = \lambda\, e^{-\lambda t}$, the hazard function is a constant and given simply by $h(t) = \lambda$; this therefore represents the simplest hazard function. When the waiting time distributions are no longer exponential, as with the cellular IMT and AT distributions, the hazard functions become time-dependent.

Since $-f(t)$ is the derivative of $S(t)$, Supplementary Equation (4) can be rewritten as

$$h(t) = -\frac{d}{dt} \log S(t). \tag{5}$$

This provides the following equation connecting just the survival and hazard functions:

$$S(t) = e^{-\int_0^t h(s)ds}. \tag{6}$$

In the case of multiple competing (say k) risks, the above equations can be generalized in a straightforward manner. The total hazard function is defined as the sum of cause-specific hazard functions $h_k(t)$,

$$h_{total}(t) = \sum_k h_k(t), \tag{7}$$

and the all-cause survival function is given by

$$S_{total}(t) = e^{-\int_0^t h_{total}(s)ds}. \tag{8}$$

The all-cause survival function in Supplementary Equation (8) gives the probability of none of the k events having occurred until time t and provides the basic equation for the competing risks analysis to be used later in the estimation of parameters of the IMT and AT distributions.

Basic introduction to copulas

Copulas (Latin for "link" or "bond"), as the name suggests, are functions that "join together" one-dimensional distribution functions to form multivariate distribution functions[7]. A more mathematical intuition for these functions can be obtained as follows: consider a pair of random variables X and Y, which could represent, for example, the times to division of two sister cells. Note that these random variables need not be independent, and indeed in the case of division times of sister cells, are highly correlated. Denote the cumulative distribution functions (cdf) of X and Y by $F(x)$ and $G(y)$, respectively, and a joint distribution $H(x,y) = \Pr(X \le x, Y \le y)$. For any pair of real numbers (x,y), three numbers can be associated with the pair: $F(x), G(y)$ and $H(x,y)$. Since all three numbers lie in the interval $[0,1]$, each pair (x,y) maps to a point $(F(x), G(y))$ in the unit square $[0,1] \times [0,1]$. This ordered pair in turn corresponds to a number $H(x,y)$ in $[0,1]$. The correspondence that assigns the value of the joint distribution function to each ordered pair of values of the individual distribution functions $F(x)$ and $G(y)$ is a function called a copula. This observation is embodied in Sklar's Theorem, which states that for a given joint distribution function $H(x,y)$ with marginals $F(x)$ and $G(y)$, there exists a copula C such that for all real (x,y)

$$H(x,y) = C(F(x), G(y)). \tag{9}$$

Conversely, if C is a copula and F and G are distribution functions, then the function H defined by Sklar's theorem in Supplementary Equation (9) is a joint distribution function with F and G as marginals. The likelihood framework developed later relies on this converse statement of Sklar's theorem. Functionally, the power of copulas becomes evident by rewriting Supplementary Equation (9) in terms of density functions (denoted by small letters) instead of the cdfs (denoted by capital letters):

$$h(x,y) = c\big(F(x), G(y)\big)\, f(x) g(y). \tag{10}$$

The copula density on the right hand side of Supplementary Equation (10) captures the correlations among the random variables X and Y, thereby separating the dependence structure from the univariate marginals. Supplementary Equation (10) will form the core

component of modeling the correlations among sister cells in the statistical algorithm developed later in this section.

How are copula functions created? There are a number of ways, including geometric methods, to construct copulas[7]. The easiest method, however, is by inverting Sklar's Theorem in Supplementary Equation (9) and using known multivariate distributions. Noting that $(F(x), G(y))$ lies in the unit square $[0,1] \times [0,1]$, we have from Supplementary Equation (9):

$$C(u, v) = H\left(F^{-1}(u), G^{-1}(v)\right), \tag{11}$$

where (u, v) lie in the unit square $[0,1] \times [0,1]$. For example, by choosing F and G to be the standard univariate normal distribution ϕ, and H to be the standard bivariate normal N_ρ (with Pearson correlation coefficient ρ), we obtain what is known as the Gaussian copula, given by $C_\rho^{Gauss}(u, v) = N_\rho(\phi^{-1}(u), \phi^{-1}(v))$. Note that using the Gaussian copula along with standard univariate normal margins in Supplementary Equation (9) would again provide the standard bivariate normal distribution.

Algorithm based on competing risks analysis and copulas to infer unbiased IMT and AT distributions

To infer the underlying (unobserved or "hidden") IMT and AT distributions that can generate the experimentally observed distributions, it is necessary to model the stochastic competition of cellular fates and the high sister correlations in IMT and AT. High sister correlations can lead to an inaccurate inference of the distribution parameters if unaccounted for, especially when IMT data is limited due to a large extent of drug-induced apoptosis. While it is challenging to model correlated data and this usually neglected for simplicity, recent work in the context of DNA methylation has highlighted the improvement in inference that can be achieved by incorporating correlations[8]. We therefore incorporated sister correlations into our algorithm, neglecting higher order lineage correlations since we found them to be smaller than sister-sister correlations. Since the Exponentially Modified Gaussian (EMG) does not have a multivariate version, copulas provide a flexible technique for modeling bivariate EMG distributions with arbitrary correlations[7]. While copula functions have been used extensively in finance[9], there exists surprisingly few applications in biological problems. Our work highlights the power of copulas, and should motivate more widespread use in biological contexts especially in the growing field of genomics and epigenetics where modeling correlated data is often important[8]. The copula framework was used in conjunction with the competing risks analysis method to develop a statistical algorithm to simultaneously infer unbiased estimates of the inter-mitotic time (IMT) and apoptosis time (AT) distributions of HCT116 colon cancer cells while accounting for the experimentally observed sister correlations. Note that this method is completely general and can be used in the analysis of any cell line with arbitrary IMT and AT distributions, treated with drugs at any concentration. The steps of the method are explained in detail below:

(a) A likelihood model was first developed to describe the data before drug dosing, where no cell death was observed. The single cell dataset comprises snapshots at every 30 minute interval, recording the fate and lineage relationship of every cell. This data was partitioned into *i* pairs of sister cells that divided before cisplatin addition, and using Supplementary Equation (10) each sister pair was ascribed a joint probability density of division given by

$$f_i\left(t_1^i, t_2^i; \boldsymbol{\theta}_{\text{div}}^{\text{bef}}\right) = c_z\left(F\left(t_1^i; \boldsymbol{\theta}_{\text{div}}^{\text{bef}}\right), F\left(t_2^i; \boldsymbol{\theta}_{\text{div}}^{\text{bef}}\right)\right) f\left(t_1^i; \boldsymbol{\theta}_{\text{div}}^{\text{bef}}\right) f\left(t_2^i; \boldsymbol{\theta}_{\text{div}}^{\text{bef}}\right). \quad (12)$$

Here *i* denotes the sister-pair, t_1^i and t_2^i denote the division times for the two sisters in pair *i* respectively (in other words, t_1^i and t_2^i represent the ages of the cells at times of division), $\boldsymbol{\theta}_{\text{div}}^{\text{bef}}$ represents the parameter vector of the function *f*, and *F* denotes the cumulative distribution for the density function *f*. Throughout this work *f* is chosen to be the EMG function based on evidence from the data (see Supplementary section 4), but any arbitrary density function can be used instead, if deemed appropriate. The subscript on $\boldsymbol{\theta}_{\text{div}}^{\text{bef}}$ denotes "division", and the superscript means "before-drug". The function c_z is the copula density for modeling the sister-sister correlation in the data. The subscript *z* in c_z refers to a single parameter in the copula which can be related to some measure of correlation in the data[7]. For elliptic copulas like the Gaussian copula, this parameter can be the Pearson correlation. For other copula families, however, measures of linear correlation cannot be used and other measures of dependence like Kendall's Tau or Spearman's rank correlation are required[7]. The final likelihood function for the entire data set (before drug dosing) then becomes

$$L\left(\boldsymbol{\theta}_{\text{div}}^{\text{bef}}, z \middle| \boldsymbol{t}\right) = \prod_i f_i\left(t_1^i, t_2^i; \boldsymbol{\theta}_{\text{div}}^{\text{bef}}\right), \quad (13)$$

where the product is over all sister pairs. Since the likelihood function involves the copula, the R package 'copula'[10] was used in the entire analysis. This likelihood function was maximized using the "MLE" command in R to obtain the maximum likelihood parameter estimate of $\boldsymbol{\theta}_{\text{div}}^{\text{bef}}$ and *z*. The parameter errors were obtained from the variance-covariance matrix derived from the Hessian matrix. Since the EMG function has three parameters, the before-drug scenario involved inferring four parameters in total. Note that the inference of the copula parameter *z* (Pearson correlation of the division time of sisters) serves as a validation of the modeling of correlations — with 80 pairs of sister cells in the pre-cisplatin scenario, the inferred value of *z* is expected to be almost identical to the Pearson correlation measured directly from the data.

(b) After addition of cisplatin, multiple fates were observed (cell division and death; the possibility of survival will be dealt with in the next section). Therefore the likelihood function to describe the data post cisplatin was a combination of the copula (modeling the correlations among times to fates of sisters) and the competing risks framework. Under this scenario, the data is described as a set of i sister pairs, where the sisters could either have been born before the time of cisplatin administration (T_d) reaching

their fate after T_d (straddling cells), or they could have been born after T_d. Since the functional form of the distribution of inter-division times and apoptosis after drug dosing was found to be best described by the EMG (see Supplementary Tables 2 and 3), $f(t;\boldsymbol{\theta})$ represents the EMG function as with the before-drug scenario. The after drug IMT and time to death density functions are defined as $f(t;\boldsymbol{\theta}_{\text{div}}^{\text{aft}})$ and $f(t;\boldsymbol{\theta}_{\text{die}}^{\text{aft}})$, respectively. The superscript denotes "after-drug". The corresponding hazard functions for division and death after drug dosing are denoted $h(t;\boldsymbol{\theta}_{\text{div}}^{\text{aft}})$ and $h(t;\boldsymbol{\theta}_{\text{die}}^{\text{aft}})$ respectively, and can be derived from the density functions via Supplementary Equation (4).

The hazard function for either of the two cells of sister pair number i that straddles the dosing event will be the hazard for division before drug dosing, and the sum of division and death hazards after drug dosing. This was mathematically implemented by defining a piece-wise function:

$$\begin{aligned} h_{straddle,}^{i}(t) &= h\left(t;\boldsymbol{\theta}_{\text{div}}^{\text{bef}}\right), & t < T_d - T_{birth}^{i} \\ &= h\left(t;\boldsymbol{\theta}_{\text{div}}^{\text{aft}}\right) + h\left(t;\boldsymbol{\theta}_{\text{die}}^{\text{aft}}\right), & t \geq T_d - T_{birth}^{i} \end{aligned} \qquad (14)$$

Note that t in Supplementary Equation (14) denotes the *time since the individual cell was born*, not the absolute time from the start of the experiment. In other words, t denotes the age of the individual cell in Supplementary Equation (14), and can be expressed as $t = T - T_{birth}^{i}$, with T denoting the absolute time since the start of the experiment and T_{birth}^{i} denoting the absolute time when the ith sister pair was born. T_d represents the absolute time when the drug was added. With the hazard function thus defined, a straddling cell's probability to survive till an age t can be computed using Supplementary Equation (8) and Supplementary Equation (14), and is given by the following survival function:

$$S^{i}(t) = \exp\left(-\left(\int_{0}^{T_d - T_{birth}^{i}} h_{straddle}^{i}(s)ds \; + \; \int_{T_d - T_{birth}^{i}}^{t} h_{straddle}^{i}(s)ds\right)\right) \qquad (15)$$

Note that this expression for the survival function is the same for both sisters in pair number i, since their hazards are identical. Noting that the cumulative distributions of the two sisters are given by $1 - S^{i}\left(t_1^{i}\right)$ and $1 - S^{i}\left(t_2^{i}\right)$ respectively (see Supplementary Equation (2)), and that the density functions for division are given by the products of survival and hazard functions, the joint density for observing both straddling sister cells of pair number i to divide is given by

$$f_i\left(t_1^{i}, t_2^{i}\right) = c_z\left(1 - S^{i}\left(t_1^{i}\right), 1 - S^{i}\left(t_2^{i}\right)\right)\; S^{i}\left(t_1^{i}\right) h\left(t_1^{i};\boldsymbol{\theta}_{\text{div}}^{\text{aft}}\right)\; S^{i}\left(t_2^{i}\right) h\left(t_2^{i};\boldsymbol{\theta}_{\text{div}}^{\text{aft}}\right). \qquad (16)$$

Supplementary Equation (16) is the analogue of Supplementary Equation (12) for cells that straddle the drug dosing event at time T_d and both eventually divide. Note that $f_i\left(t_1^{i}, t_2^{i}\right)$ is parametrized by $\boldsymbol{\theta}_{\text{div}}^{\text{bef}}, \boldsymbol{\theta}_{\text{div}}^{\text{aft}}$ and $\boldsymbol{\theta}_{\text{die}}^{\text{aft}}$ – we dropped the parameters on the

left hand side of Supplementary Equation (16) simply for notational convenience. To reduce the number of parameters that need to be simultaneously inferred, we fixed the values of $\boldsymbol{\theta}_{\text{div}}^{\text{bef}}$ to those obtained from maximum likelihood estimation from the pre-cisplatin dataset, and inferred only the six parameters $\boldsymbol{\theta}_{\text{div}}^{\text{aft}}$ and $\boldsymbol{\theta}_{\text{die}}^{\text{aft}}$ from the post-cisplatin dataset. We also fixed the value of *z* to that calculated directly from the data (separate values of *z* for cell division and death). The next section discusses a seventh parameter – the probability of a cell being in a state of cell cycle arrest, which was also inferred along with the six mentioned here.

Sister cells that straddle or are born after T_d are more likely to die due to the effect of cisplatin. Hence along with Supplementary Equation (16), similar equations were developed for all the other possibilities, including discordant fates among the sister cells. The only modifications necessary for such cases is changing the hazard functions on the right hand side of Supplementary Equation (16) to the appropriate hazard functions that describe the eventual fate of the sister cells. For cells that were born after T_d, the total hazard function is $h(t; \boldsymbol{\theta}_{\text{div}}^{\text{aft}}) + h(t; \boldsymbol{\theta}_{\text{die}}^{\text{aft}})$, and the corresponding survival function can be obtained from Supplementary Equation (8). Finally, the total likelihood of the post-cisplatin data analogous to Supplementary Equation (13) is a product of the joint density given in Supplementary Equation (16) and all such joint densities describing all possible combinations of fates, over all i sister pairs of cells. For sister pairs with discordant cell fates, we used the simplifying condition of no correlations between the sisters and set the copula term to 1, denoting independence.

Modeling the probability of surviving treatment

Several cells survive until the end of the lineage-tracing experiment. These cells may simply have been censored (they would have divided or died had more time been allowed to elapse), or they may have been under cell cycle arrest due to the action of cisplatin. The older a surviving cell was at the end of the experiment, the more likely it is that the cell was maintained in an arrested state. Conversely, the younger a cell was when the experiment was ended, the more likely it is that that particular cell was not given enough time to undergo a fate. This physically intuitive observation was incorporated into the likelihood framework discussed above using a variable q, which denotes the probability of a surviving cell in the dataset to have been in a state of cell cycle arrest[11]. Therefore, for a sister pair that straddled T_d and both cells eventually divided, the joint density given in Supplementary Equation (16) was multiplied by the factor $(1-q)^2$, denoting that neither cell was under cell cycle arrest. For a cell that survived till the end of the experiment, its density function would be given by $q + (1 - q)S^i(t_1^i)$: the cell could have been arrested or not arrested; if not arrested with probability $(1-q)$, then $S^i(t_1^i)$ quantifies the probability that the cell survived at least till age t_1^i given the competing risk scenario. The appropriate function of q was multiplied with each of the i pair of sisters, and the final complete likelihood constructed. This final likelihood could not be maximized using standard maximization techniques, and a Metropolis Hastings MCMC algorithm was used to generate posterior distributions of all the seven parameters to be

inferred — three each from $\boldsymbol{\theta}_{div}^{aft}$ and $\boldsymbol{\theta}_{die}^{aft}$, and the probability of arrest q. The MCMC results are presented later.

Modeling delayed response of cells after drug administration

Previous work has suggested that certain drugs may take time to act on cells after being added to the *in vitro* medium[11]. This delay in drug action could in principle affect the estimation of the parameters from the last sections. To account for this possible effect, we introduced a new parameter (to be inferred from the data via the likelihood algorithm), T_{delay}. Physically, the introduction of this term delays the moment when the hazard of a cell switches from just division to division and death. Mathematically, T_{delay} adds a delay to the time when the hazard function switches in Supplementary Equation (14). As a result, the piece-wise function is changed to

$$\begin{aligned} h_{straddle,}^{i}(t) &= h\left(t;\boldsymbol{\theta}_{\mathrm{div}}^{\mathrm{bef}}\right), && t < T_d + T_{delay} - T_{birth}^{i} \\ &= h\left(t;\boldsymbol{\theta}_{\mathrm{div}}^{\mathrm{aft}}\right) + h\left(t;\boldsymbol{\theta}_{\mathrm{die}}^{\mathrm{aft}}\right), && t \geq T_d + T_{delay} - T_{birth}^{i} \end{aligned} \quad (17)$$

Similarly, the limits of integration in Supplementary Equation (15) are also changed from $T_d - T_{birth}^{i}$ to $T_d + T_{delay} - T_{birth}^{i}$. This additional term T_{delay} therefore effectively models the delay in action of the drug.

Accounting for sister correlations improves parameter estimation of the IMT distribution

Before using our full computational algorithm of the copulas combined with competing risks to analyze the data, we tested the improvement of parameter inference that can be achieved by accounting for correlations among sister cells. To this end, we used two complementary methods:

Method (1): In this approach, we assumed that the true underlying IMT distribution is represented by a dataset comprising only one cell per lineage, which ensures that there are no correlations in this dataset. Therefore, parameters of the distribution inferred from this uncorrelated dataset will represent the 'true' underlying parameters. Correlated datasets of similar size (obtained by analyzing sister pairs) can then be used to test the usefulness of our copula-based inference approach – our approach based on the correlated data should provide inferred parameters closer to the 'true' parameters as compared to inference using the standard method of non-linear least squares (NLS). Specifically, by randomly choosing one cell division time from each lineage in the experimental dataset, we obtained 41 inter-mitotic times and inferred the parameters of this IMT distribution ('true' parameters). To obtain a correlated dataset of similar size, we randomly chose 20 sister pairs out of the 80 sister pairs in our full dataset. We then used NLS as well as our copula-based approach to infer parameters from the correlated dataset. We compared the closeness of each of the inferred parameters i ($i = \mu, \sigma, \lambda$) to the 'true' parameters using the square of a simple distance metric

$D_{NLS,i} = inferred\ parameter\ from\ NLS_i - true\ parameter_i$; $D_{copula,i} = inferred\ parameter\ from\ copula\ method_i - true\ parameter_i$.

Since the 41 independent samples and the 20 sister pairs can be chosen in many ways, we performed this entire analysis 1,000 times, each time drawing a different random set of data and computing the closeness of the parameter inferences to the 'true' values. We found that our copula-approach consistently outperformed the NLS approach, doing better 64.3% of the time for μ, 56.3% of the time for σ, and 61.1% of the time for λ. These values did not change when we increased the sampling from 1,000 to 1,500 and 2,000 times, ruling out the possibility that the higher frequency of improved parameter estimates occurred by chance.

While the above analysis highlights the improvement achieved by our method, it provides an under-estimate of the improvement that could potentially be reached. This fact arises because the 'true' parameters, inferred from a dataset of just 41 samples, do not perfectly represent the real underlying distribution due to the small sample size, and different realizations of drawing 41 independent division times produce slightly different distributions. This leads to an increased chance of the NLS method having better performance than our copula method in specific instances. Therefore, while this method provides a way of demonstrating the importance of determining correlations directly from the data, we investigated a second method in which the truth is unequivocally known.

Method 2: In this method, we used simulations to generate correlated random number pairs (representing sister cells) from an underlying distribution. Hence by construction we know the underlying true distribution. We chose an EMG distribution with the same parameters that described our pre-cisplatin IMT data (μ = 28.576, σ = 2.453, λ = 0.274). We then used the NLS method as well as our copula method to infer the distribution parameters from this correlated dataset. As with Method 1, we calculated the distance of parameter estimates from both NLS and copula methods to the true parameter values ($D_{NLS,i}$ and $D_{copula,i}$, respectively) which were used to generate the data. This procedure was repeated $\sim$1,000 times. When we used 80 simulated sister pairs, a number chosen to match our experimental data, we found that our method was closer to the truth 61.8% of the time for μ, 71.1% of the time for σ, and 66.9% of the time for λ. We repeated the entire analysis for 100 simulated sister pairs and found that our method does better 62.4% of the time for μ, 71.4% of the time for σ, and 67.3% of the time for λ. In addition, since in this method we have precise knowledge of the true underlying parameters, we also checked the magnitude of improvement in parameter estimation achieved by our copula method over the NLS method. To do this, we defined an improvement metric D_i $(i = \mu, \sigma, \lambda)$ given by $D_i = |D_{NLS,i}| - |D_{copula,i}|/true\, parameter_i$ for each of the three parameters, whenever $|D_{NLS,i}| > |D_{copula,i}|$. This metric provides a measure of how close the copula inference is to the true parameters compared to the NLS inference. While we found that there was only $\sim 1\%$ median improvement for μ, the improvement in estimation of the other two parameters was very large: $\sim 14.6\%$ median, 23.4% 3rd quartile improvement for σ and $\sim 12\%$ median, 26% 3rd quartile improvement for λ (see Supplementary Figure **4** for the distributions of D_i).

Results from the statistical algorithm — maximum likelihood and MCMC simulations

We will now discuss results from the application of the statistical algorithm described in the previous sections to single cell lineage tracing data of HCT116 cells. Results of the maximum likelihood analysis on pre-cisplatin data are presented in Supplementary Table 4, and results from the MCMC analysis of the post-cisplatin data are shown in Supplementary Table 5. Finally, results from analysis of the post-cisplatin data with time delay in drug action are displayed in Supplementary Table 6.

Supplementary Table 4: Inferred parameters for the pre-cisplatin cell division events using Maximum Likelihood. μ, σ, λ correspond to the parameters of the IMT distribution and z is the copula parameter (i.e. the Pearson correlation for a Gaussian copula).

Parameter	Maximum Likelihood estimate	Standard deviation
μ	28.6	0.5
σ	2.4	0.3
λ	0.27	0.04
z	0.71	0.05

Supplementary Table 5: Inferred parameters of IMT and AT distributions post-cisplatin using MCMC simulations.

Parameter	Mean of posterior distribution	Standard deviation of posterior distribution
μ (division)	27.7	0.8
σ (division)	1.5	0.7
λ (division)	0.014	0.001
μ (death)	58	7
σ (death)	29	3
λ (death)	0.5	0.4
q	0.27	0.02

Supplementary Table 6: Inferred parameters of IMT and AT distributions post-cisplatin for the model with a time delay in drug action. Results were obtained using MCMC simulations and are almost identical to those in Supplementary Table 5, indicating that T_{delay} has a negligible effect.

Parameter	Mean of posterior distribution	Standard deviation of posterior distribution
μ (division)	27.6	0.8
σ (division)	1.4	0.8
λ (division)	0.012	0.001
μ (death)	58	7
σ (death)	29	4

$\boldsymbol{\lambda}$ **(death)**	0.5	0.4
$\boldsymbol{q}$	0.27	0.02
$\boldsymbol{T_{delay}}$	0.1	0.1

6. Age-dependent birth-death process model

Basic model with no circadian coupling

To validate the results of the inference procedure and explore the mechanistic origins of the observed correlations among cellular lineages, a computational model based on birth-death processes was developed to mimic the single cell lineage tracing experiments. Cellular proliferation was modeled such that a cell divides or dies based on probabilistic rules. In particular, each cell division results in exactly two daughters being born and death removes one cell from the population. We used a version of this model that keeps track of the age of every single extant cell[12]. The probability per unit time of division or death depends on the cell's age in a manner that reproduces the correct, non-exponential functional form (EMG in this case) of these distributions. In brief, a kinetic Monte Carlo simulation was performed where the acceptance probability of any event (division or death) for a cisplatin treated cell depends on the exponential factor $\exp\left[-\left(h(t;\boldsymbol{\theta}_{\mathrm{div}}^{\mathrm{aft}}) + h(t;\boldsymbol{\theta}_{\mathrm{die}}^{\mathrm{aft}})\right)\Delta t\right]$, where t is the age of the cell, $h(t;\boldsymbol{\theta}_{\mathrm{div}}^{\mathrm{aft}})$ and $h(t;\boldsymbol{\theta}_{\mathrm{die}}^{\mathrm{aft}})$ are the hazard functions discussed in Supplementary section 5, and Δt is the time step for the simulations. Once an event is destined to occur, the choice between division or death for that cell is generated based on the ratio $h(t;\boldsymbol{\theta}_{\mathrm{div}}^{\mathrm{aft}}) / \left(h(t;\boldsymbol{\theta}_{\mathrm{div}}^{\mathrm{aft}}) + h(t;\boldsymbol{\theta}_{\mathrm{die}}^{\mathrm{aft}})\right)$. For cellular events before addition of the drug, only the hazard function $h(t;\boldsymbol{\theta}_{\mathrm{div}}^{\mathrm{bef}})$ was used. Details of the general simulation procedure are given in [12]. The time step Δt was chosen such that it was much smaller than the average times of the IMT and time to death distributions. Throughout this work $\Delta t = 0.1$ frames ($= 3$ minutes) was used.

In addition, the birth-death process simulations described above were implemented on a directed graph, such that the vertices or nodes of the graph represent individual cells and edges represent mother-daughter relationships. Growing the cellular population on a graph allows for tracking of lineage relationships between every cell in the population, extant or dead. The absolute time of birth, time of fate, and type of fate (division or death) were recorded as vertex attributes. Initial conditions for all simulations were 30 ancestor cells (similar to the HCT116 single cell dataset), whose ages were randomly chosen from a uniform distribution on the interval $[0, M]$, where M is the average of the IMT distribution before cisplatin administration (parameters given in Supplementary Table 4). The progeny of these 30 ancestor cells were tracked over time starting from $T = 0$. All simulations were performed using the R package "igraph"[13].

Similar to the single cell lineage tracking experiment data, the initial 30 ancestor cells were first allowed to proliferate to $\sim 250 - 290$ cells (~ 3 generations) in the absence of drug. The absence of drug was modeled by setting $\boldsymbol{\theta}_{\mathrm{div}}^{\mathrm{bef}}$, the EMG parameters for division in the absence of cisplatin, from Supplementary Table 4. After proliferating to ~ 250 cells, the parameters

were changed and set using the results in Supplementary Table 5 to reflect addition of cisplatin, and further proliferation was simulated. Division times before and after drug addition were calculated from the recorded vertex attributes, allowing computation of the histograms and correlations shown in the main text.

Modeling cell fate dependence on stochastic protein production/degradation
To investigate the dependence of cell fate on stochastically fluctuating levels of one or multiple proteins, we developed a computational model where, in addition to the basic single cell lineage generating mechanism (described above), we simulated protein production with rate k_{prod} and degradation with rate k_{deg} within each single cell over time. To this end we used the standard approach of generating a uniformly distributed random number between 0 and 1 for each cell at each time step and comparing it with the quantities $[\text{Protein}]k_{\text{deg}}\Delta t$ and $[\text{Protein}]k_{\text{deg}}\Delta t + k_{\text{prod}}\Delta t$ to decide which reaction will occur, if any. We developed two models: one in which the concentration of one protein (Protein X) controls the cell division probability, and one in which the concentrations of two proteins (Protein X and Protein Y) control the cell division probability. The concentrations of the proteins in a mother cell at the time of division are kept identical to the concentrations inherited by the two daughters. These inherited concentrations are then coupled to the hazard functions of the cells to control cell fate probabilities. Since the goal of these models was to investigate whether the cousin-mother inequality could be recapitulated, using data from our pre-cisplatin study, we modeled only division and neglected cell death. Finally, for both these models, we studied different mixing properties of the proteins: for the case when the protein is 'mixing', i.e. when it loses memory of its level over time scales that are shorter than a single cell division time, we chose the production/degradation parameters $k_{\text{prod}} = 2.5\ \text{frame}^{-1}$ and $k_{\text{deg}} = 0.05\ \text{frame}^{-1}$. This choice allows for large fluctuations in the protein levels over time. For the case when the protein is 'non-mixing' and loses memory over time scales much larger than a single cell's lifetime, the parameters were chosen to be $k_{\text{prod}} = 0.05\ \text{frame}^{-1}$ and $k_{\text{deg}} = 0.005\ \text{frame}^{-1}$. This choice ensured that the protein level a cell was born with hardly changes by the time that cell divides. The mathematical details of the two models are as follows:

<u>Protein X model</u>: As the Protein X concentration at the time at which the mother divides increases, the parameter μ for the two daughters' hazard functions also increases, thereby enhancing the probability of longer division times for the two daughter cells. If the Protein X concentration in the mother decreases, there is an increased probability of shorter divisions for the daughters. Mathematically, this was achieved by setting $\mu = \mu_0 + 0.65\ [\text{Protein X}] - 25$ for every cell, where $[\text{Protein X}]$ represents the Protein X concentration in a cell at the time it was born. The concentration of Protein X in the 30 ancestor cells were randomly chosen from [10,50] (arbitrary units). This parametrization allowed us to maintain the correct magnitude of the sister correlations as observed in our pre-cisplatin dataset. The remainder of the parameters of the model were kept the same as inferred for the pre-cisplatin case (Supplementary Table 4): $\mu_0 = 28.5, \sigma = 2.4, \lambda = 0.27$, while k_{prod} and k_{deg} were chosen as described above.

Protein X and Protein Y model: To allow the division probability of each cell to depend on the levels of two proteins, we generated stochastic simulations of two independent proteins X and Y. When the ratio of their levels X/Y increases beyond 1, the daughters are more likely to divide more slowly while they are more likely to divide faster if the ratio of the protein levels is smaller than 1. This effect was achieved by coupling the hazard functions of each cell to the protein levels: $\mu = \mu_0 + 14.0\ ([\text{Protein X}]/[\text{Protein Y}] - 1)$ when both X and Y are non-mixing; $\mu = \mu_0 + 6.0\ ([\text{Protein X}]/[\text{Protein Y}] - 1)$ when X is mixing and Y is non-mixing; $\mu = \mu_0 + 20.0\ ([\text{Protein X}]/[\text{Protein Y}] - 1)$ when both X and Y are mixing. As with the Protein X only case, these parametrizations were chosen so as to reproduce the experimentally observed sister correlations. The remainder of the parameters of the model were kept the same as inferred for the pre-cisplatin case (Supplementary Table 4): $\mu_0 = 28.5, \sigma = 2.4, \lambda = 0.27$, while k_{prod} and k_{deg} were chosen as described above.

Modeling circadian gating of the cell cycle

The circadian clock was ascribed a period of 24 hours (48 frames) as found in experiments using the HCT116 cell line[14]. The phase of the clock Φ in the model was determined by the absolute time: $\Phi = \frac{2\pi}{48}\ T$ (for convenience, all units of time used in the equations are frames, not hours). To model gating of the cell cycle by the circadian clock, the probability for division of a cell was chosen based on the clock phase at which the cell was born — cells born at certain phases of the clock would have a higher probability of dividing at lower ages than cells born at other phases of the clock. Mathematically, this was achieved by making the parameter μ of the EMG a sinusoidal function of the clock phase at cell birth. As mentioned in Supplementary section 4, the mean of the EMG is given by $\mu + 1/\lambda$, while the variance is given by $\sigma^2 + 1/\lambda^2$. Therefore, by changing μ, the mean of the IMT distribution and hence the hazard of division can be changed without affecting the variance. The following general structure for μ was used to introduce gating of the cell cycle for any individual cell:

$$\mu = \mu_0 + A \sin(\Phi), \tag{18}$$

where Φ is the clock phase at time of birth of that particular cell. Sister cells are born at the same clock phase (for simulations relaxing this assumption, see below), cousins at similar phases, and mother-daughter pairs at potentially very different phases, depending on the length of the cell cycle compared to the clock period. For the simulations incorporating circadian gating, an extra vertex attribute recording Φ for each cell was added. The crucial aspect to note regarding the two free parameters μ_0 and A in Supplementary Equation ((18), is that they need to be chosen in a way that reproduces the IMT distributions defined by $\boldsymbol{\theta}_{\text{div}}^{\text{bef}}$ (for the pre-cisplatin division events) and $\boldsymbol{\theta}_{\text{div}}^{\text{aft}}$ (for the post-cisplatin division events). Note that the EMG distributions, whose parameters $\boldsymbol{\theta}_{\text{div}}^{\text{bef}}$ and $\boldsymbol{\theta}_{\text{div}}^{\text{aft}}$ were inferred using the statistical algorithm in Supplementary section 5, represent the true underlying (though unobserved in the case of $\boldsymbol{\theta}_{\text{div}}^{\text{aft}}$) distributions from which the observed division and death data must have been generated. Therefore, in order to recapitulate the distributions of the *observed* data in the experiment, the model must use as underlying distributions EMG functions with $\boldsymbol{\theta}_{\text{div}}^{\text{bef}}$ and $\boldsymbol{\theta}_{\text{div}}^{\text{aft}}$ as parameters. Therefore μ_0 and A in Supplementary Equation ((18) must be chosen in a way

that the pre-competition IMT distribution is similar to the distributions parameterized by $\boldsymbol{\theta}_{\mathrm{div}}^{\mathrm{bef}}$ and $\boldsymbol{\theta}_{\mathrm{div}}^{\mathrm{aft}}$. Furthermore, not all combinations of parameters μ_0 and A that recapitulate the underlying distributions can recapitulate the observed correlations in the division times. Hence this poses an additional constraint on the choice of parameterization of Supplementary Equation ((18). In order to satisfy both these constraints simultaneously, the following parameterization was used in all simulations incorporating gating of the cell-cycle: $\boldsymbol{\theta}_{\mathrm{div,circadian}}^{\mathrm{bef}} \rightarrow (\mu^i = 28.7 + 4.8\sin(\Phi^i), \sigma = 2.2, \lambda = 0.85)$ for the pre-cisplatin scenario and $\boldsymbol{\theta}_{\mathrm{div,circadian}}^{\mathrm{aft}} \rightarrow (\mu^i = 31.0 + 13.0\sin(\Phi^i), \sigma = 0.01, \lambda = 0.02)$ for the post-cisplatin scenario. The superscript i denotes the sister pair number, as in Supplementary section 5. Therefore, for the pre-cisplatin scenario, the circadian model to describe IMT distributions and correlations in IMT has four free parameters $\mu_0, A, \sigma, \lambda$. Since the EMG function describing the IMT distribution requires 3 parameters for complete characterization, our model requires *only* one extra free parameter to also explain the entire IMT correlation structure pre-cisplatin. Note that there may exist other choices of parameter values for $\mu_0, A, \sigma, \lambda$ that explain the data equally well — the choice given above is only meant to serve as an example of how a minimal model with circadian gating can explain the single cell lineage tracing data. The number of free parameters for the post-cisplatin scenario is discussed below. Finally, note that the definition of the clock phase based on the absolute time implies that all cells have their circadian clocks synchronized. This is a simplifying assumption and usually not true in bulk populations of cells. However, this assumption does not affect the experimental observations this model aims to explain – correlations within *lineages of single cells*. This point is explained in more detail with supporting simulations below.

The simulations using the circadian gating model were started with an initial condition of 30 cells with randomly drawn ages between 0 and the mean of the inferred IMT distribution (parameters in Supplementary Table 4). The initial absolute time was set to $T = 0$ and as before, the time step Δt was chosen to be $\Delta t = 0.1$ frames ($= 3$ minutes). The lineages of each of the 30 original cells were then followed over time.

Varying the periods of the oscillator gating of the cell cycle

Besides a 24hour circadian period for the oscillator gating of the cell cycle, a number of other periods were tested for their ability to reproduce the cousin-mother inequality observed in the pre-cisplatin HCT116 lineage data. The parameters $\sigma = 2.2$, $\lambda = 0.85$ were kept unchanged across all of these simulations. Only the parameters μ_0 and A were tuned to reproduce the correct sister correlations and the IMT distributions observed in the data. Supplementary Table 7 provides all parameter values that were used to generate Fig. 6 and Supplementary Figure 10. Parameters for the period 24 hours (48 frames) are given in the preceding paragraph.

Supplementary Table 7: Parameters used in simulations with varying time periods of the oscillator gating the cell cycle. The parameters were chosen to reproduce the sister correlations and IMT distribution observed in the pre-cisplatin data. Note that 1 frame = 0.5 hours.

Time Period (frames)	μ_0	A
**7**	31.0	4.0
**12**	30.0	4.2
**14**	32.0	5.0
**21**	31.0	4.2
**24**	31.0	4.2
**28**	32.7	5.5
**37**	28.7	4.8
**72**	31.0	4.2
**96**	30.5	4.0

Gating of apoptosis by the circadian clock

Gating of apoptosis was incorporated in a manner similar to Supplementary Equation ((18), with an identical underlying principle — cells born at certain phases of the circadian clock are more likely to die at lower ages as compared to other cells. With gating of two pathways (division and death), an additional phase difference between the two gated pathways must be considered. This phase difference was accounted for by using the variable $\Delta\varphi$ in the following manner for defining μ of the time to death distribution:

$$\mu = \mu_0 + A\sin(\Phi + \Delta\varphi) \quad (19)$$

Similar constraints as with Supplementary Equation ((18) need to be satisfied by the two free parameters μ_0 and A in Supplementary Equation (19). The parameterization chosen was $\boldsymbol{\theta}_{\text{die,circadian}}^{\text{aft}} \rightarrow \left(\mu^i = 57 + 30.0\sin\left(\Phi^i + \Delta\varphi\right), \sigma = 20.2, \lambda = 0.62\right)$. For the post-cisplatin scenario, therefore, there were eight free parameters required to describe all correlations in IMT and AT as well as the full IMT and AT distributions: a set of four parameters $\mu_0, A, \sigma, \lambda$ representing $\boldsymbol{\theta}_{\text{div,circadian}}^{\text{aft}}$, and another set of four $\mu_0, A, \sigma, \lambda$ representing $\boldsymbol{\theta}_{\text{die,circadian}}^{\text{aft}}$. Note that since three parameters each were required to characterize the post-cisplatin IMT and AT distributions, only two additional parameters were required by our model to capture the entire correlation structures in post-cisplatin IMT and AT. Finally**,** various values of $\Delta\varphi$ between 0 and 2π were explored, and $\Delta\varphi \sim 0$ was found to best explain the data, suggesting that gating of cell cycle and cell death pathways must occur approximately in phase. These parameters for the time to death distribution along with the parameters for the IMT distribution (given above) together explain all experimentally observed IMT and AT distributions as well as the correlations before and after cisplatin treatment.

Random phase change of the circadian clock during cell division

The model for circadian gating described above makes the simplifying assumption that the circadian clocks of all cells in the population are synchronized. This assumption is usually not true in a bulk population where the individual clocks are poorly synchronized, unless treated with dexamethasone or serum shocked[15]. The single cell lineage tracing experiment analyzed in this work was performed under standard cell culture conditions, and hence the cells would not be expected to show synchronicity *at the bulk level*. However, when the oscillations of circadian proteins in progeny emerging from a *single cell* are tracked, the phase of the clocks in the two daughters after division faithfully start close to the phase value where the mother cell ended[15–17]. Hence, over a few generations (which is the duration of the entire experiment studied here), the cells *within one particular lineage* would have circadian clocks that are synchronized to a high degree. Since the model developed here aims to explain correlations between family members within a lineage, the simplifying assumption of synchronized clocks is a very good approximation. Having said that, small phase shifts have been noticed at the time of division, presumably due to stochastic distribution of circadian proteins from mother to daughter cells[15,17]. To ensure that these small phase shifts do not qualitatively change any of this work's results, simulations were performed including random phase shifts in the clock phase at birth of daughter cells (Supplementary Figure 8). This therefore ensures that the two daughters at the time of birth have slightly different clock phases. The random phase shift was chosen uniformly between $[0, P]$. All results shown in the main text remained almost quantitatively the same even with P as high as $\pi/_6$, as shown in Supplementary Figure 8. Only with $P \geq \pi/_2$ do the correlations among sisters and cousins show a noticeable decrease.

Random circadian phases for different cellular lineages

As discussed above, the results of the simulations shown in the main text were derived under the assumption that all cells across different lineages had synchronized circadian clocks. This assumption was made purely for the sake of simplicity of the model and for reducing computing time and memory requirements as we needed to keep track of the phases of each lineage separately over time. This assumption was not meant to be physically realistic, but captured the minimal requirement that sisters have similar circadian phases. Since different lineages are independent in birth-death models such as the one we use in this study, our results would not change if we assumed different phases across lineages.

To demonstrate the validity of our results shown in the main text regarding the lineage correlation, in particular the cousin-mother inequality, even in the absence of this assumption, we developed a modified model: we chose the circadian phase of the 30 starting cells randomly from a uniform distribution, $[0, 2\pi]$. As a result, the phases of cells across lineages are no longer synchronized at any later time (Supplementary Figure 9a). We found that our original results for the lineage correlations still hold in this modified version of the model (Supplementary Figure 9b), proving that as long as the sister cells have similar circadian phases, the experimentally observed correlation structures can be quantitatively reproduced by our model. Note that in this modified model, the cells within a lineage still have synchronized circadian clocks, but we showed in the previous subsection that adding small amounts of randomness in the passing of phase from mother to daughter does not change our results.

Modeling correlated cell fates of sisters

In addition to the correlation structures in times to fate, very high similarities between the eventual fates of the sister and cousin cells were also observed (Supplementary Figure 2a). To check whether these results could also be accounted for in the birth-death simulation framework, the circadian gating model was updated based on our experimental observation that p53 dynamics of sisters is correlated and associated with cellular fate (Fig. 2e,f). Two additions were made to the simulation procedure: (1) In the step where the decision to undergo an event (either division or death) for every extant cell is made depending on comparisons of independent uniform random numbers between 0 and 1 with the exponential factors $\exp[-\left(h\left(t;\boldsymbol{\theta}_{\mathrm{div}}^{\mathrm{aft}}\right)+h\left(t;\boldsymbol{\theta}_{\mathrm{die}}^{\mathrm{aft}}\right)\right)\Delta t]$, correlated random numbers were introduced for the sisters. Sister cells among the extant cells were identified in each time step and correlated uniform random numbers between 0 and 1 were generated from a bivariate Gaussian copula, using the "copula" package in R. Independent random numbers were generated for all the other non-sister cells. (2) A similar method for generating correlated uniform random numbers for sisters was used for the fate-determining step. Once an event is destined to occur, the choice between division or death was generated based on comparisons of uniform random numbers to the ratios $h\left(t;\boldsymbol{\theta}_{\mathrm{div}}^{\mathrm{aft}}\right)/\left(h\left(t;\boldsymbol{\theta}_{\mathrm{div}}^{\mathrm{aft}}\right)+h\left(t;\boldsymbol{\theta}_{\mathrm{die}}^{\mathrm{aft}}\right)\right)$ for each cell destined to a fate at that time step. Instead of using independent random numbers, correlated random numbers were generated for the sisters. In both the steps, the same value of correlation was used, thereby adding only one extra free parameter overall. A Pearson correlation of 0.95 was used for the random numbers to generate Supplementary Figure 13. The similarity in fates (Supplementary Figure 13a) was calculated from the simulations as follows: We kept track of the number of cells N_{ext} that were extant in the simulation at the time the hazard functions were switched to mimic cisplatin addition. Among these N_{ext} cells, N_{sis} and N_{cous} were the number of sisters and cousins, respectively. We then tracked the fates (division or death) of these N_{ext} cells after cisplatin addition. N_{div} cells divided and N_{die} cells died, from among the N_{ext} cells. We then counted how many of the N_{div} cells that divided, were sister and cousin pairs, defined as $N_{sis,div}$ and $N_{cous,div}$ respectively. Similarly, we defined $N_{sis,die}$ and $N_{cous,die}$ from the N_{die} cells that died. The probability of sisters sharing the same fate after cisplatin treatment was then found as $P_{sis}=(N_{sis,div}+N_{sis,die})/N_{sis}$, and similarly the probability of cousins sharing the same fate was $P_{cous}=(N_{cous,div}+N_{cous,die})/N_{cous}$. The probability of two random cell pairs exhibiting the same fate was calculated as $P_{rand}=(N_{div}/N_{ext})^2+(N_{die}/N_{ext})^2$. We found that in our simulations P_{rand} was always approximately 50%, while P_{sis} and P_{cous} were higher, as shown in Supplementary Figure 13a.

7. Supplemental experimental methods

Live cell microscopy for cell cycle analysis

To track cell cycle stage we incorporated a Cerulean-hGem lentiviral reporter into HCT116 p53-VKI cells. Approximately 10,000 cells were plated to poly-D-lysine coated glass bottom dishes (MatTek corporation) in McCoy's media with 10% FBS. Cells were grown for 48 hours prior to

imaging to allow cells to attach. Prior to imaging, media was replaced with RPMI without phenol red or riboflavin and supplemented with 5% FBS (clear RPMI) to minimize background fluorescence. Cells were imaged every 30 minutes for 24 hours to identify cell cycle stage, then media was replaced with premixed clear RPMI-media + 12.5 µM cisplatin and cells were imaged for an additional 72 hours without media replacement.

Data analysis

Cells were tracked with custom ImageJ scripts using the phase channel. Custom Matlab (Mathworks) scripts were used to subtract the background from images using the rolling ball algorithm (Sternberg 1983) and acquire single cell trajectories of p53-Venus and Cerulean-hGem levels. Code available upon request. Cell-cycle stage was determined by visual inspection of Cer-hGem levels.

References

1. Sandler, O. *et al.* Lineage correlations of single cell division time as a probe of cell-cycle dynamics. *Nature* **519,** 468–471 (2015).
2. Bhuyan, B. K. & Groppi, V. E. Cell cycle specific inhibitors. *Pharmacol. Ther.* **42,** 307–348 (1989).
3. Paek, A. L., Liu, J. C., Loewer, A., Forrester, W. C. & Lahav, G. Cell-to-Cell Variation in p53 Dynamics Leads to Fractional Killing. *Cell* **165,** 631–642 (2016).
4. Sakaue-Sawano, A. *et al.* Visualizing spatiotemporal dynamics of multicellular cell-cycle progression. *Cell* **132,** 487–98 (2008).
5. Johmura, Y. *et al.* Necessary and sufficient role for a mitosis skip in senescence induction. *Mol. Cell* **55,** 73–84 (2014).
6. *Model Selection and Multimodel Inference - A Practical | Kenneth P. Burnham | Springer*.
7. *An Introduction to Copulas | Roger B. Nelsen | Springer*.
8. Jenkinson, G., Pujadas, E., Goutsias, J. & Feinberg, A. P. Potential energy landscapes identify the information-theoretic nature of the epigenome. *Nat. Genet.* **49,** 719–729 (2017).
9. Bouyé, E., Durrleman, V., Nikeghbali, A., Riboulet, G. & Roncalli, T. *Copulas for Finance - A Reading Guide and Some Applications*. (Social Science Research Network, 2000).
10. Hofert, M., Kojadinovic, I., Maechler, M. & Yan, and J. *copula: Multivariate Dependence with Copulas*. (2017).
11. Tyson, D. R., Garbett, S. P., Frick, P. L. & Quaranta, V. Fractional proliferation: a method to deconvolve cell population dynamics from single-cell data. *Nat. Methods* **9,** 923–928 (2012).
12. Stukalin, E. B., Aifuwa, I., Kim, J. S., Wirtz, D. & Sun, S. X. Age-dependent stochastic models for understanding population fluctuations in continuously cultured cells. *J. R. Soc. Interface* **10,** 20130325 (2013).
13. Tamas Nepusz, G. C. The igraph software package for complex network research. *InterJournal* **Complex Systems,** (2006).

14. Relógio, A. *et al.* Ras-Mediated Deregulation of the Circadian Clock in Cancer. *PLoS Genet.* **10,** (2014).
15. Nagoshi, E. *et al.* Circadian gene expression in individual fibroblasts: cell-autonomous and self-sustained oscillators pass time to daughter cells. *Cell* **119,** 693–705 (2004).
16. Feillet, C. *et al.* Phase locking and multiple oscillating attractors for the coupled mammalian clock and cell cycle. *Proc. Natl. Acad. Sci.* **111,** 9828–9833 (2014).
17. Bieler, J. *et al.* Robust synchronization of coupled circadian and cell cycle oscillators in single mammalian cells. *Mol. Syst. Biol.* **10,** 739 (2014).

SUPPLEMENTARY FIGURES

Fig. S1

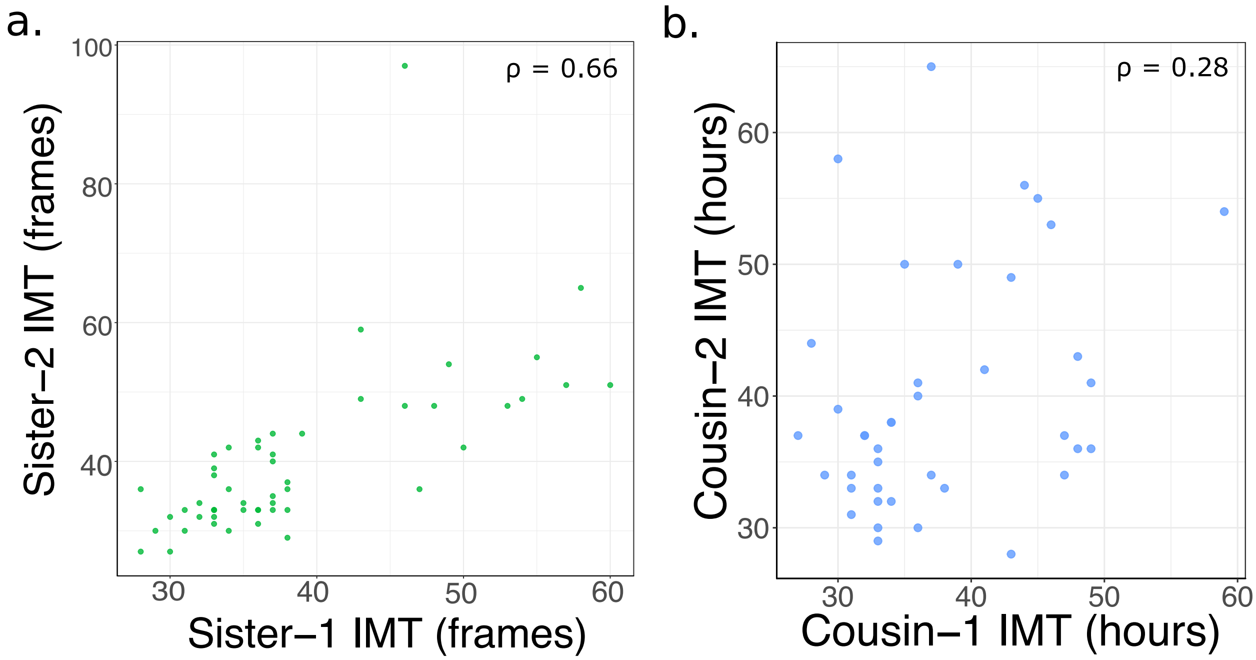

Supplementary Figure 1: *Lineage correlations in IMT of cells that straddle the cisplatin dosing event.* (a) Correlation in sisters and (b) correlation among cousins. The Pearson correlation (ρ) in each case is mentioned on top of each panel.

Fig. S2

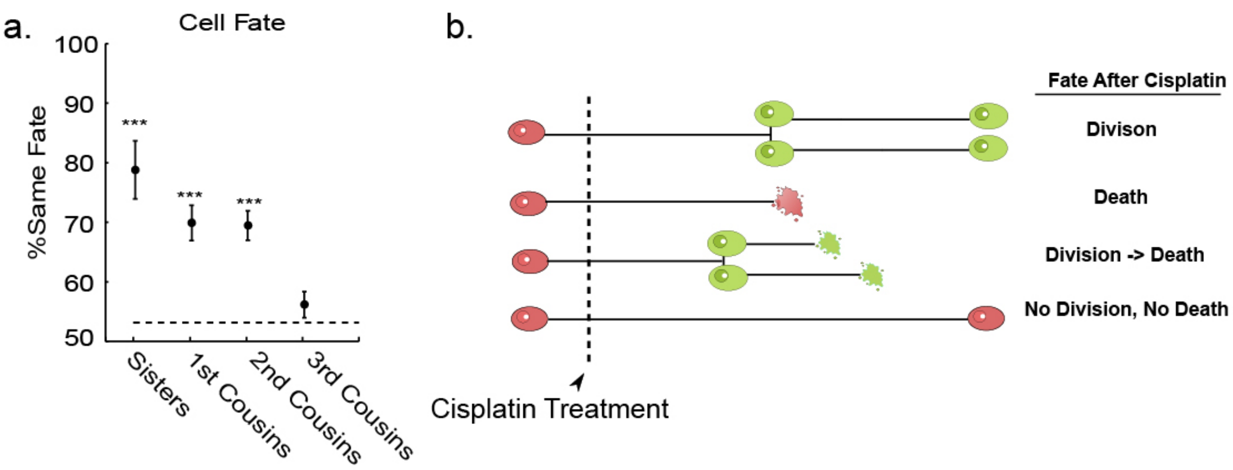

c.

Fate of Cell	% Cells	%Same Fate: Indep. Events	Sisters	1st Cousins	2nd Cousins	3rd Cousins
Division	27%	7%	22%	17%	20%	17%
Death	28%	8%	24%	14%	7%	5%
Division->Death	36%	13%	30%	29%	32%	30%
No Division, No Death	9%	1%	5%	4%	2%	0%
Total	100%	29%	80%	64%	61%	49%

Supplementary Figure 2: *Cell fate correlations between cells within a lineage.* (a) Percentage of related cell pairs that share the same fate (death or survival only). The dashed line is the percentage of unrelated cells that share the same fate (52%) *** P < .001 error bars. See Methods section for error bars and calculation of significance (b) Four different cell fates are possible after cisplatin treatment as cells can either divide or die, do both or do neither. (c) Percentage of cell pairs by relationship that had the same cell fate after cisplatin treatment. The probability of any two cells sharing the same fate is shown for comparison.

Fig. S3

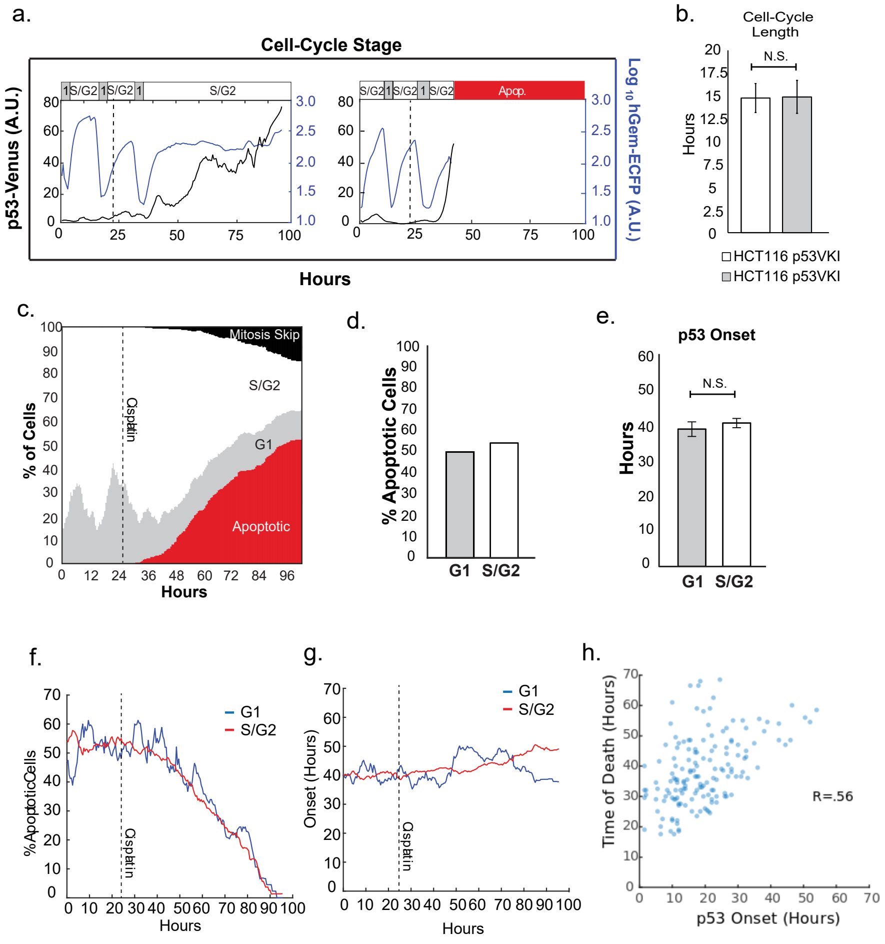

i.

Fate of Cell	% of Cells	% Fate G1	% Fate S/G2
Division	38%	24%	76%
Death	19%	67%	33%
Division->Death	34%	15%	85%
No Division, No Death	9%	83%	17%
Total	100%	34%	66%

Supplementary Figure 3: *Cell cycle stage does not alter p53 dynamics or cell fate in response to cisplatin.* (a) Representative traces of p53 Venus and Cer-hGem in single cells. Cell cycle stage is at the top of the graph and was determined by Cer-hGem levels and the timing of the previous cell division. 1 represents G1. (b) Cell cycle length was measured using time-lapse microscopy in HCT116 p53 VKI cells with and without the cell cycle reporter Cer-hGem. N > 200 cells for each cell line. (c) Percentage of apoptotic, G1, S/G2 cells and cells that underwent a mitosis skip at each time point after 12.5 μM cisplatin. N = 341 cells. (d-e) Cells separated by their cell cycle stage at the time of cisplatin addition. Neither the percentage of apoptotic cells (d) nor p53 onset (e) differ between cells in G1 and cells in S/G2. (f) The percentage of apoptotic cells for cells in G1 or S/G2 phases during each time point after cisplatin treatment. (g) The time of p53

onset for cells in G1 or S/G2 phase during each time point after cisplatin treatment. (h) The time of death (y-axis) for different the p53 onset time (x-axis). Note that time of death here is defined from the moment cisplatin was added. R (pearson's correlation coefficient) (i) The percentage of cells in each cell-cycle stage that had one of the four fates outlined in Supplementary Figure 2C. Error bars in (b) and (e) represent the standard error of the mean. A t-test was used to test for significance.

Fig. S4

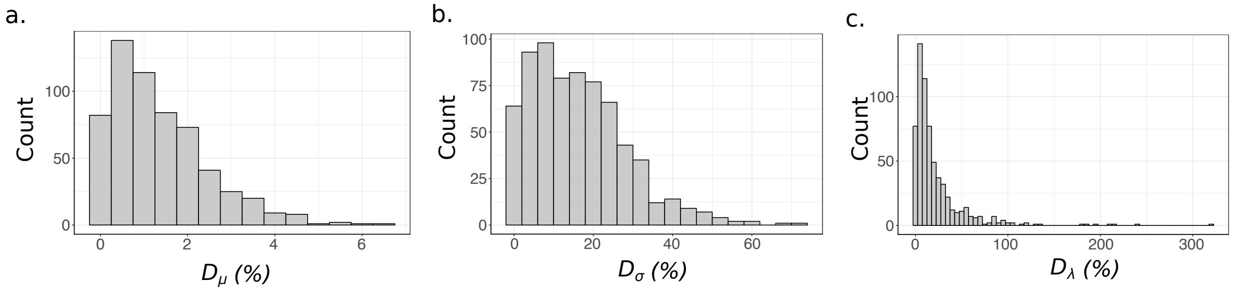

Supplementary Figure 4: *Accounting for sister correlations improves the inference of the underlying IMT distribution.* (a)-(c) Percentage improvement in the inferred parameters μ, σ and λ after accounting for sister correlations via the copula framework. The metrics D_i $(i = \mu, \sigma, \lambda)$ are defined as $D_i = |D_{NLS,i}| - |D_{copula,i}|/true\ parameter_i$ for each of the three parameters, whenever $|D_{NLS,i}| > |D_{copula,i}|$. For more details, see Supplementary section 5.

Fig. S5

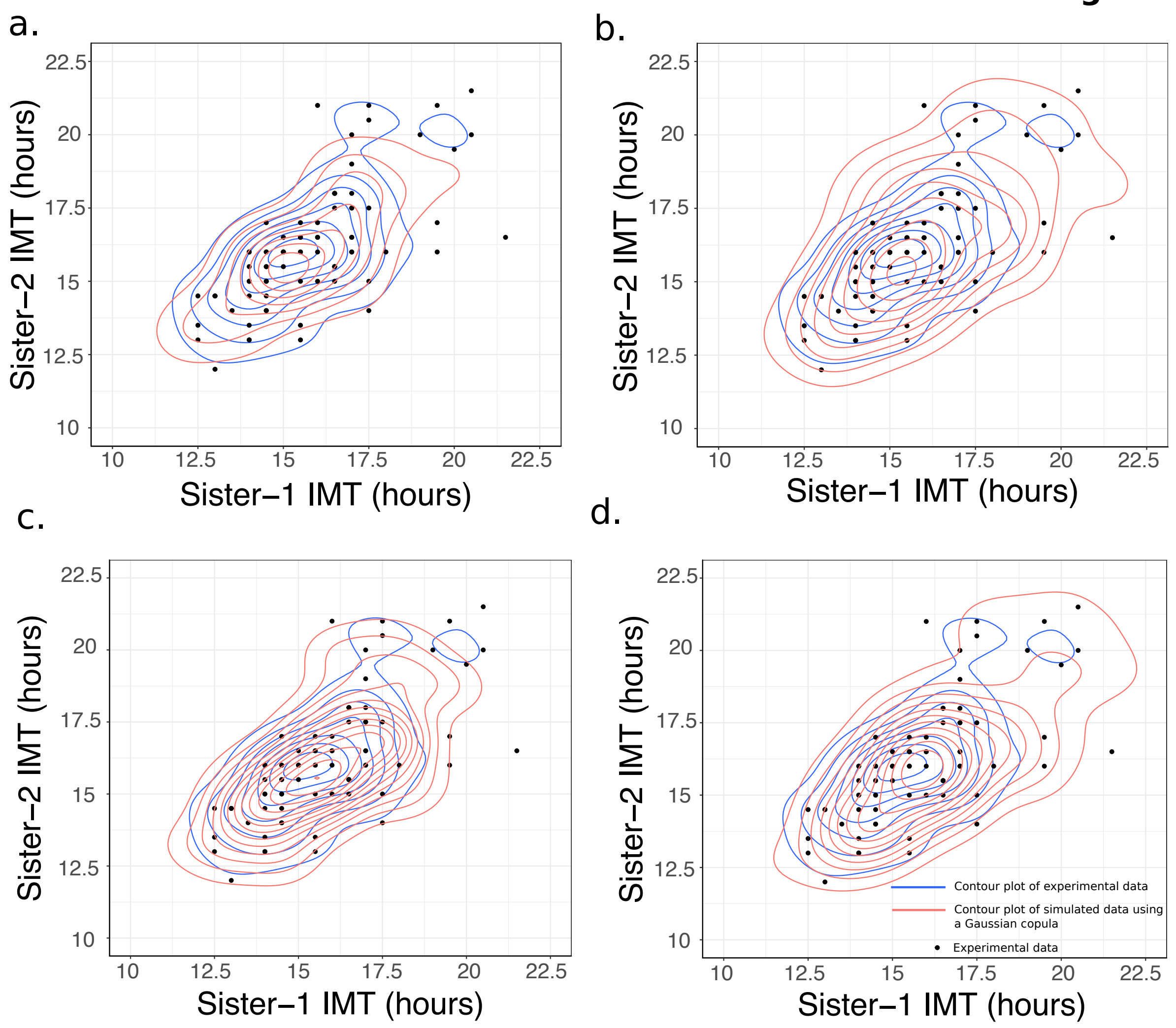

Supplementary Figure 5: *The copula formulation captures the bivariate sister IMT distribution.* (a)-(d) Contour plots of the bivariate density obtained from four independent sets of simulated data with 80 sister pairs, a number chosen to match the number of sisters in the experimental data. In the simulated datasets, the univariate margins were chosen to be the inferred EMG distribution as given in Supplementary Table 4, and the Pearson correlation was set to the inferred value given in Supplementary Table 4.

Fig. S6

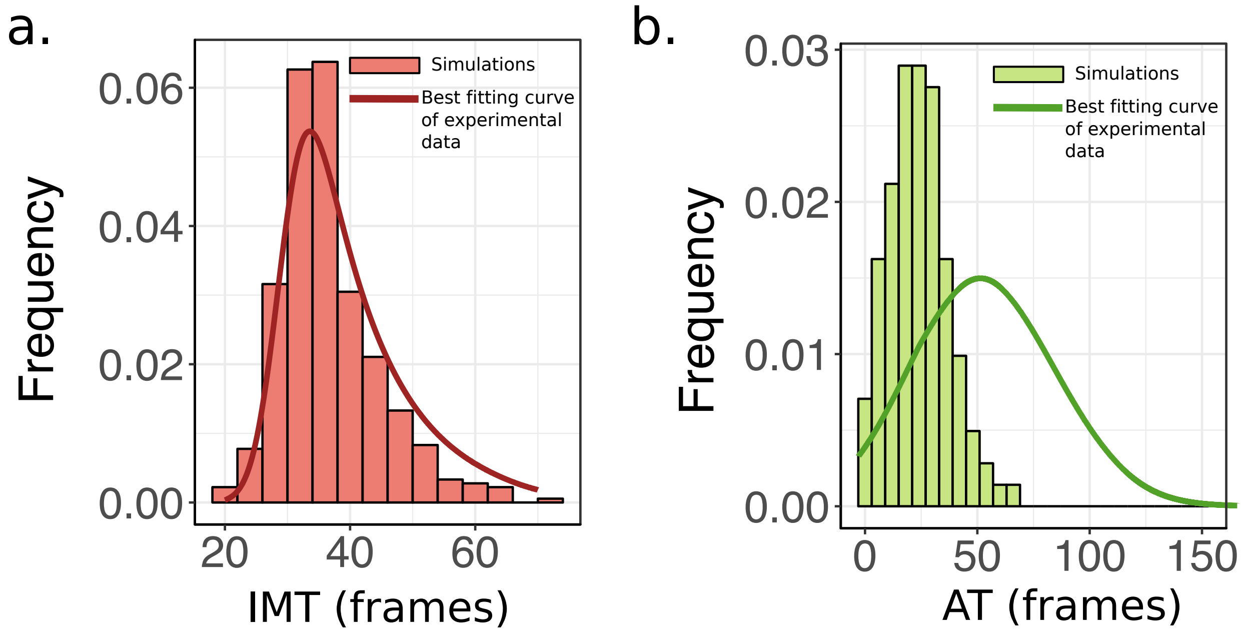

Supplementary Figure 6: *The measured (experimentally observed) IMT and AT distributions do not represent the true underlying distributions.* Histograms in (a), (b) represent output distributions resulting from using the experimentally observed IMT and AT distributions (solid lines) as inputs to our birth-death process model simulations. Stochastic competition between cell division and death skews the output distributions (histograms), such that they are biased and no longer match the observed distributions (solid lines). This observation highlights the importance of inferring the correct hidden IMT and AT distributions using our computational algorithm detailed in Supplementary section 5.

Fig. S7

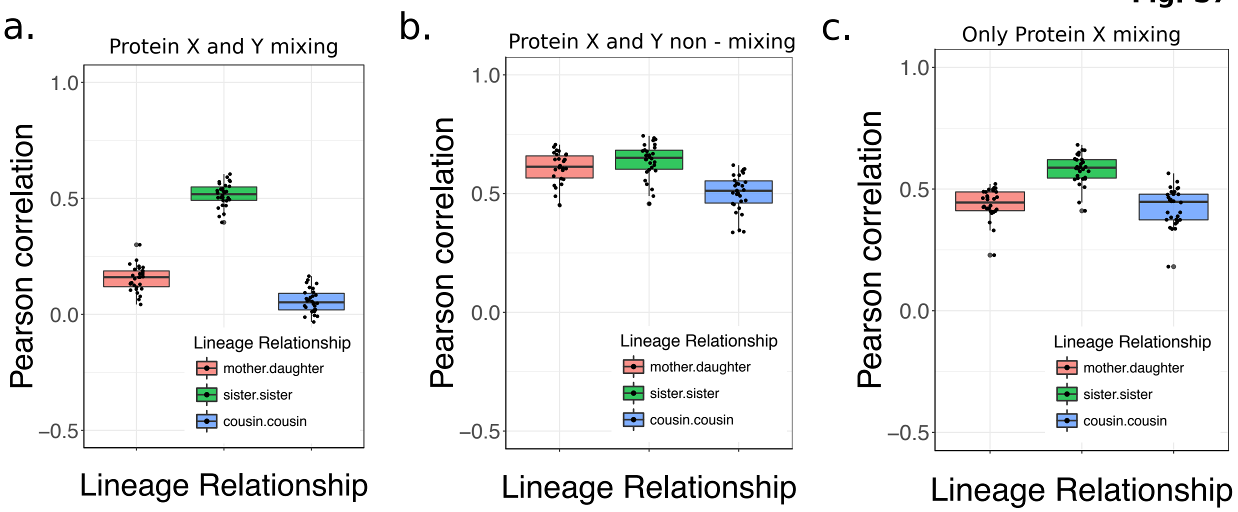

Supplementary Figure 7: *The cousin-mother inequality cannot be explained by a stochastic protein production degradation model in which cell fate is controlled by two proteins, X and Y.* (a)-(c) Lineage correlations obtained from simulations of stochastic protein production and degradation combined with the single cell birth-death process. (a) Both proteins X and Y are mixing, thereby losing memory of the initial protein levels a mother passes on to the daughter cells; (b) neither X nor Y are mixing, and hence retain memory of the initial protein levels a cell is born with; (c) only protein X is mixing. As can be seen in the three panels, the cousin-mother inequality cannot be recapitulated in any case. In addition, in (b) and (c), the mother-daughter correlations become very large, inconsistent with our experimental observations. All boxplots represent the 1st, 2nd and 3rd quartiles of the lineage correlations generated from 30 simulation runs.

Fig. S8

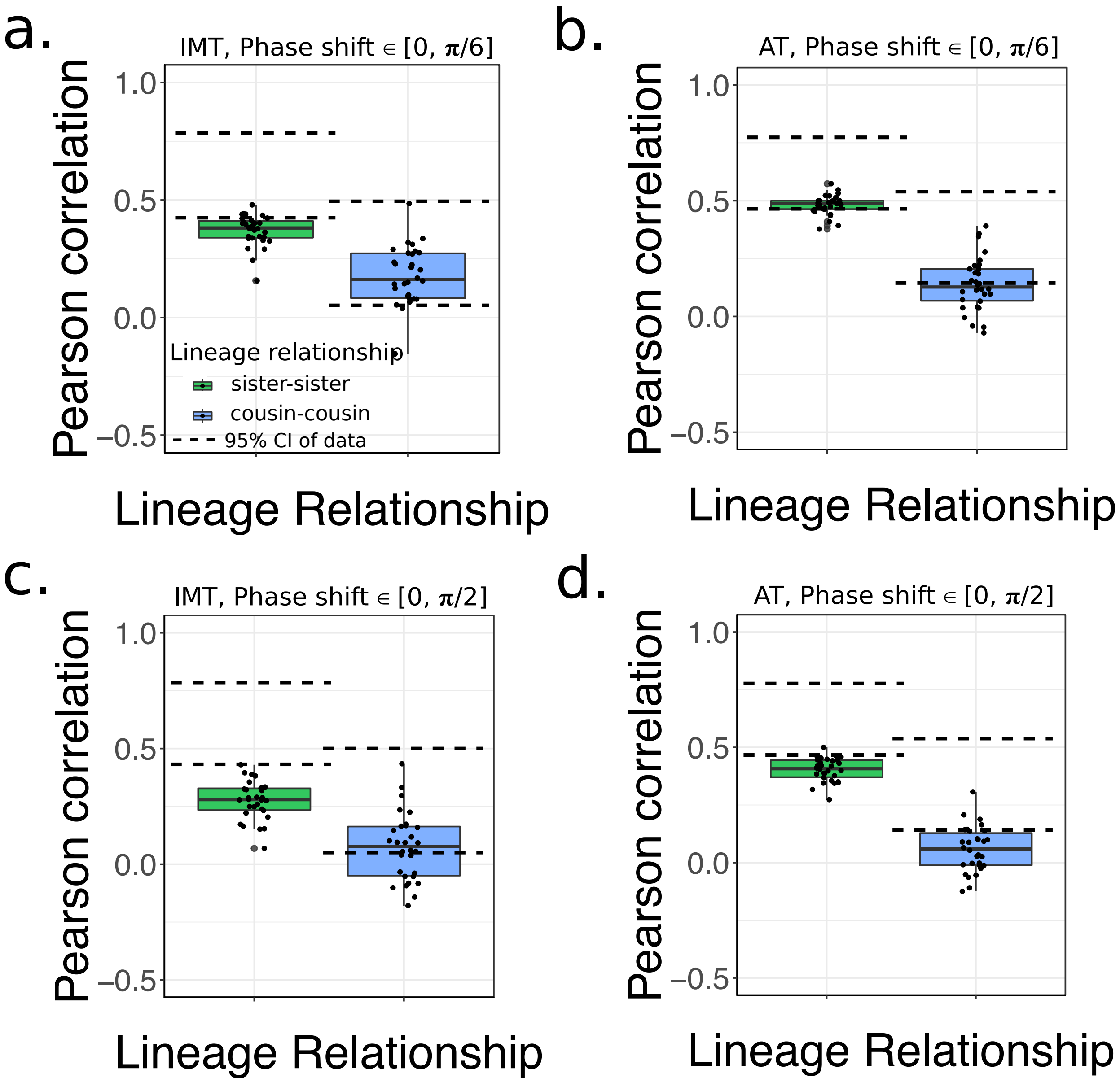

Supplementary Figure 8: *Random phase shifts of the circadian clock between sisters at time of birth do not affect the post-cisplatin correlation structures.* (a-b) Correlations in IMT and AT, respectively, show no noticeable change when random phase shifts between $[0, \pi/6]$ are added to the sister circadian clocks at birth, compared to the results in Fig. 5b,d of the main text. (c-d) Lineage correlations decreased only when very large phase shifts between $[0, \pi/2]$ were added. Details of the simulation procedure are given in Supplementary section 6. All boxplots represent the 1st, 2nd and 3rd quartiles of the lineage correlations generated from 30 simulation runs.

Fig. S9

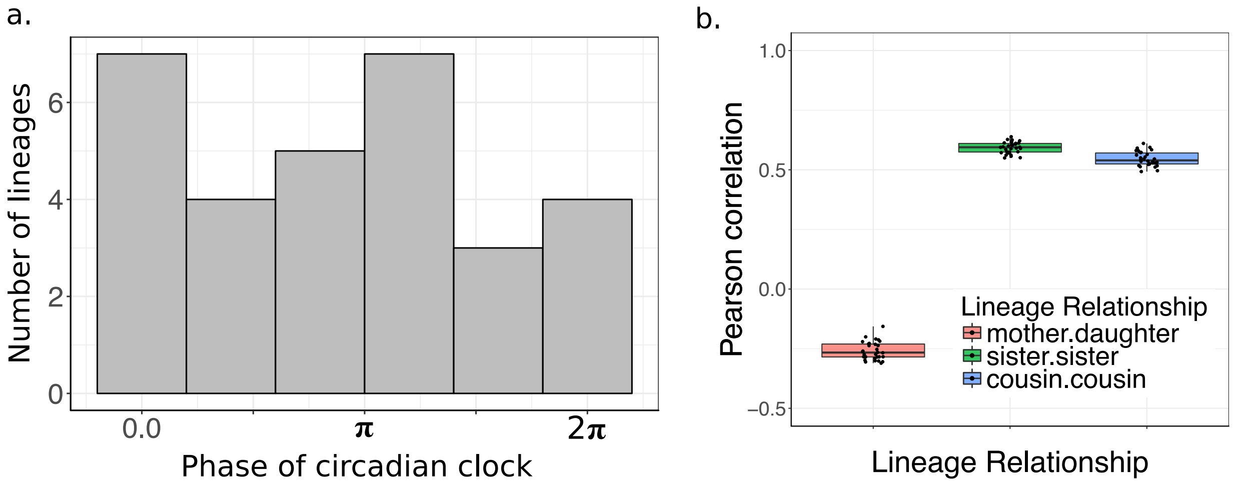

Supplementary Figure 9: *Circadian phases of cells across lineages do not have to be synchronized to recapitulate the observed correlation structure.* (a) Distribution of the phase of the circadian clock across different lineages. Cells in different lineages have randomly different phases in this model, but cells within a given lineage are synchronized. (b) Lineage correlations generated by the model in (a) recapitulate the experimental data. The boxplot represents the 1st, 2nd and 3rd quartiles of the lineage correlations generated from 30 simulation runs.

Fig. S10

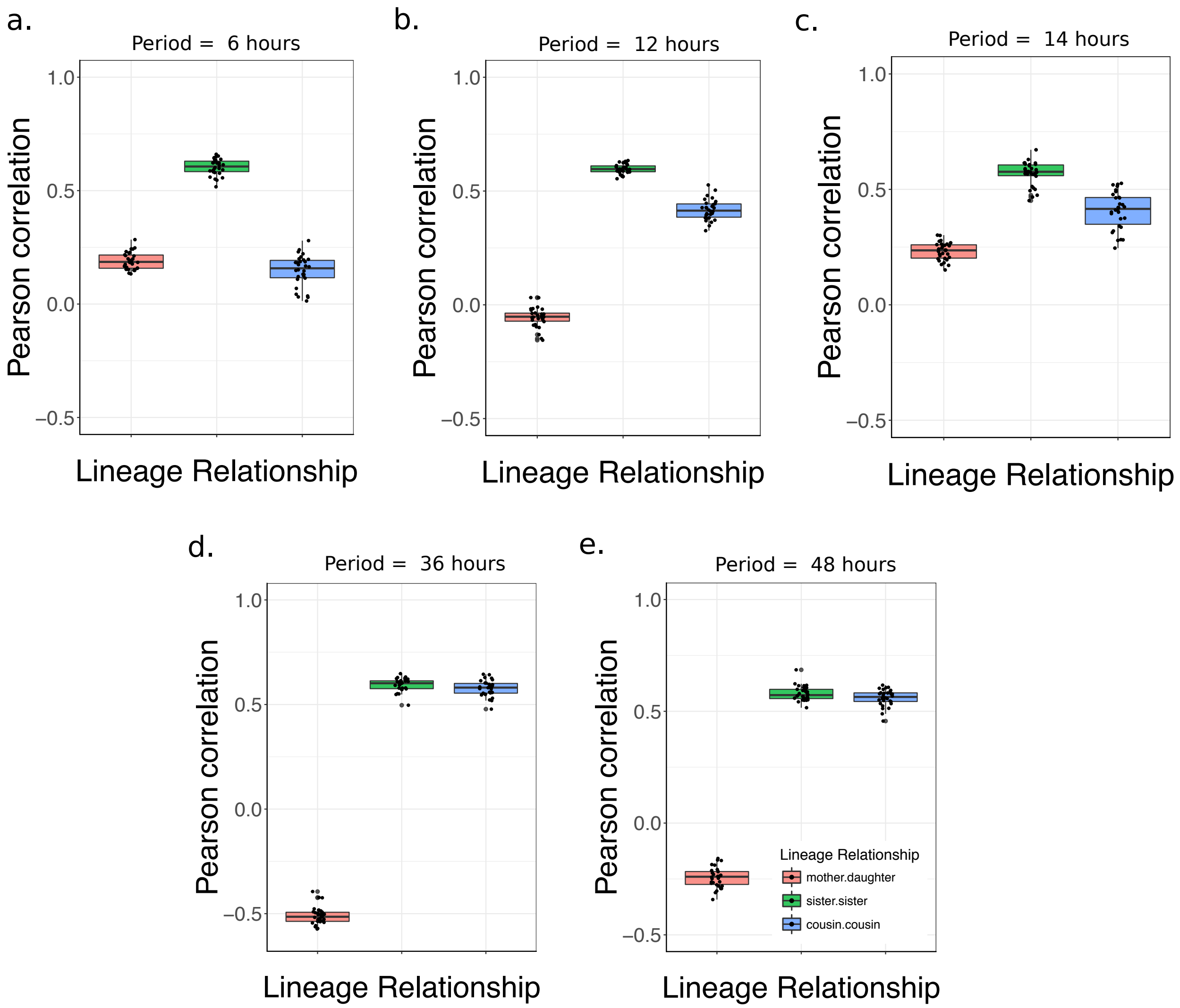

Supplementary Figure 10: *The cousin-mother inequality can be reproduced only for certain values of the oscillator time period.* (a)-(e) Lineage correlations obtained from simulations in which the time period of the oscillator was varied. Only certain multiples of approximately 12 hour time periods were able to recapitulate the data, for example 12 and 48 hours (24 hours as well; these results are shown in Fig. 5f), but not 36 hours. Parameters used for generating these plots are given in Supplementary section 6. All boxplots represent the 1st, 2nd and 3rd quartiles of the lineage correlations generated from 30 simulation runs.

Fig. S11

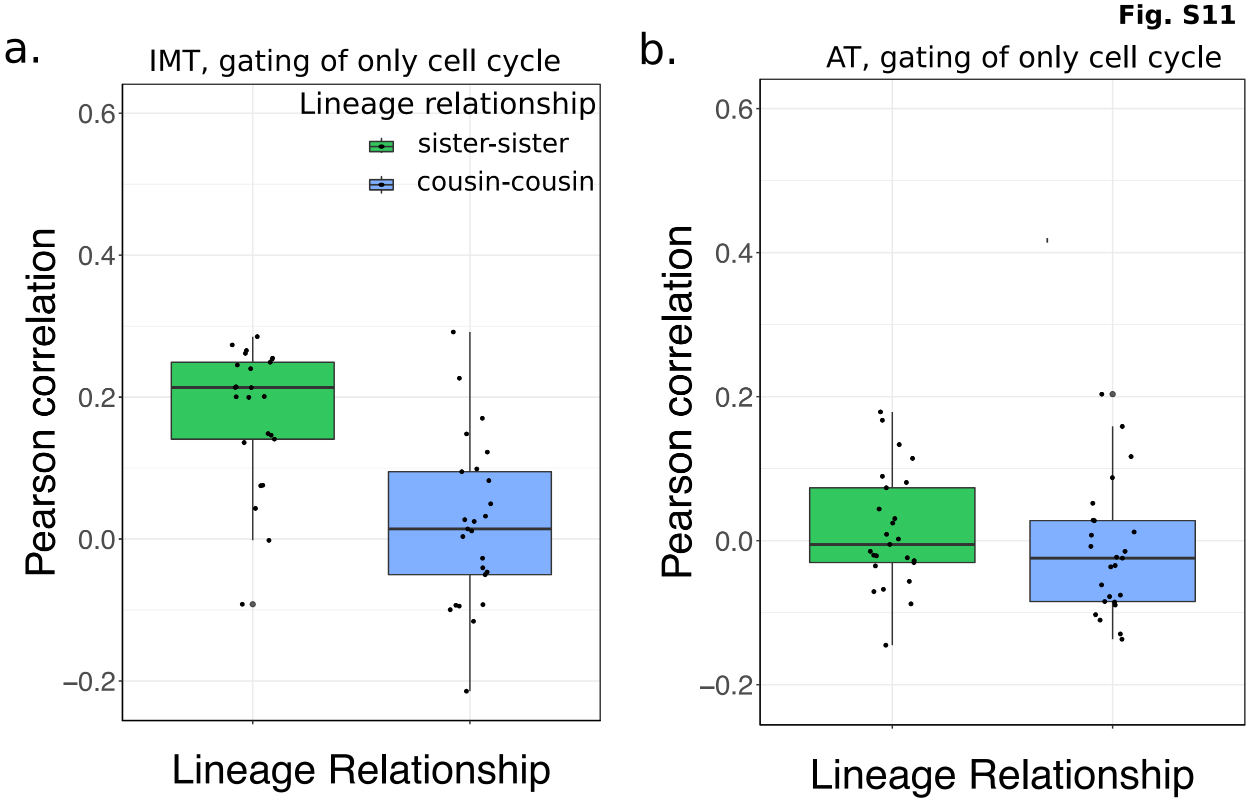

Supplementary Figure 11: *Gating of only the cell cycle cannot explain post-cisplatin correlation structures***.** (a-b) Lineage correlations in the post-cisplatin scenario, when only circadian gating of the cell cycle, and not cell death, was simulated. The input IMT distribution for the simulations with circadian gating was given by $\boldsymbol{\theta}^{\mathrm{aft}}_{\mathrm{div,circadian}}$ (see Supplementary section 6). The input AT distribution was given by the inferred parameters for death from Supplementary Table 5. These parameter choices reproduce the experimentally observed IMT and AT distributions, but not the lineage correlation structures, showing the importance of gating the death pathways. All boxplots represent the 1st, 2nd and 3rd quartiles of the lineage correlations generated from 25 simulation runs.

Fig. S12

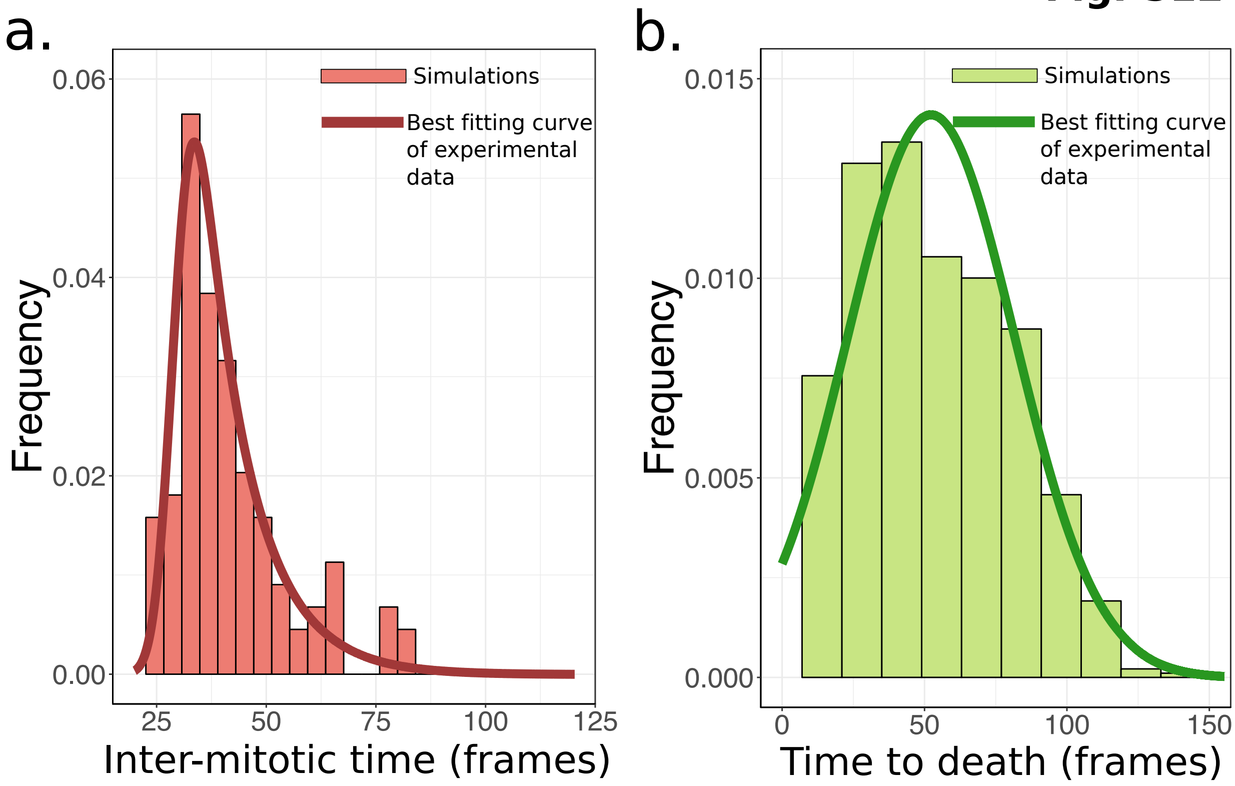

Supplementary Figure 12: *The circadian gating model recapitulates the experimentally observed IMT and AT distributions in the post-cisplatin scenario.* (a) The observed IMT and (b) AT distributions are reproduced by the theory. The histograms represent the post-competition output of our circadian gating simulations. Solid lines represent the observed experimental data. IMT and AT input distributions for the simulation are parameterized by $\boldsymbol{\theta}^{\mathrm{aft}}_{\mathrm{div,circadian}}$ and $\boldsymbol{\theta}^{\mathrm{aft}}_{\mathrm{die,circadian}}$ respectively (see Supplementary section 6 for details). Note that 1 frame = 0.5 hours.

Fig. S13

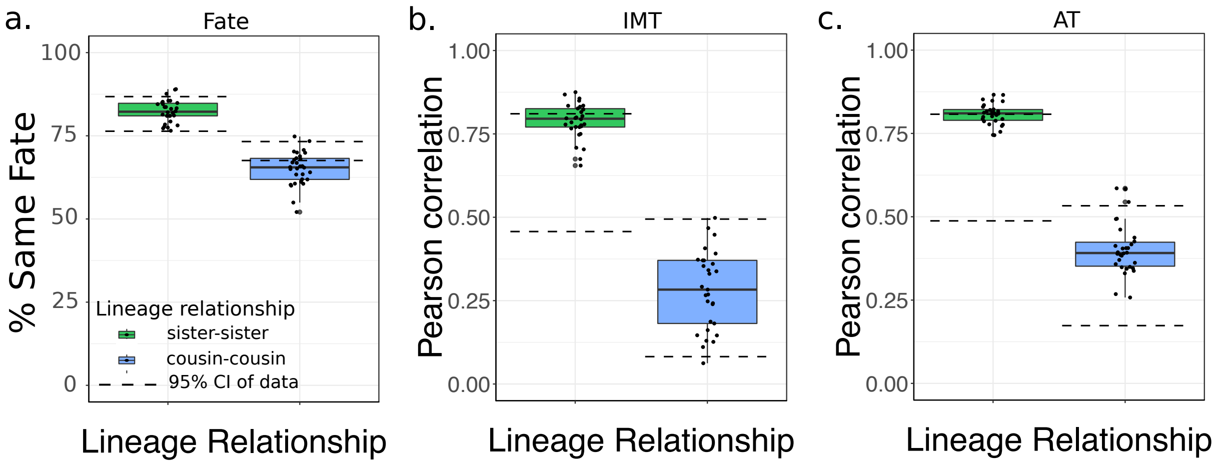

Supplementary Figure 13: *The circadian gating model recapitulates similarities in cell fates after cisplatin treatment.* (a) Similarities in cell fates among sisters and cousins as generated by our simulations. (b-c) The theory simultaneously recapitulates all the IMT and AT correlations as well. For details of the simulation procedure and parameters, see Supplementary section 6. All boxplots represent the 1st, 2nd and 3rd quartiles generated from 30 simulation runs.

Supplementary Figure 14-1

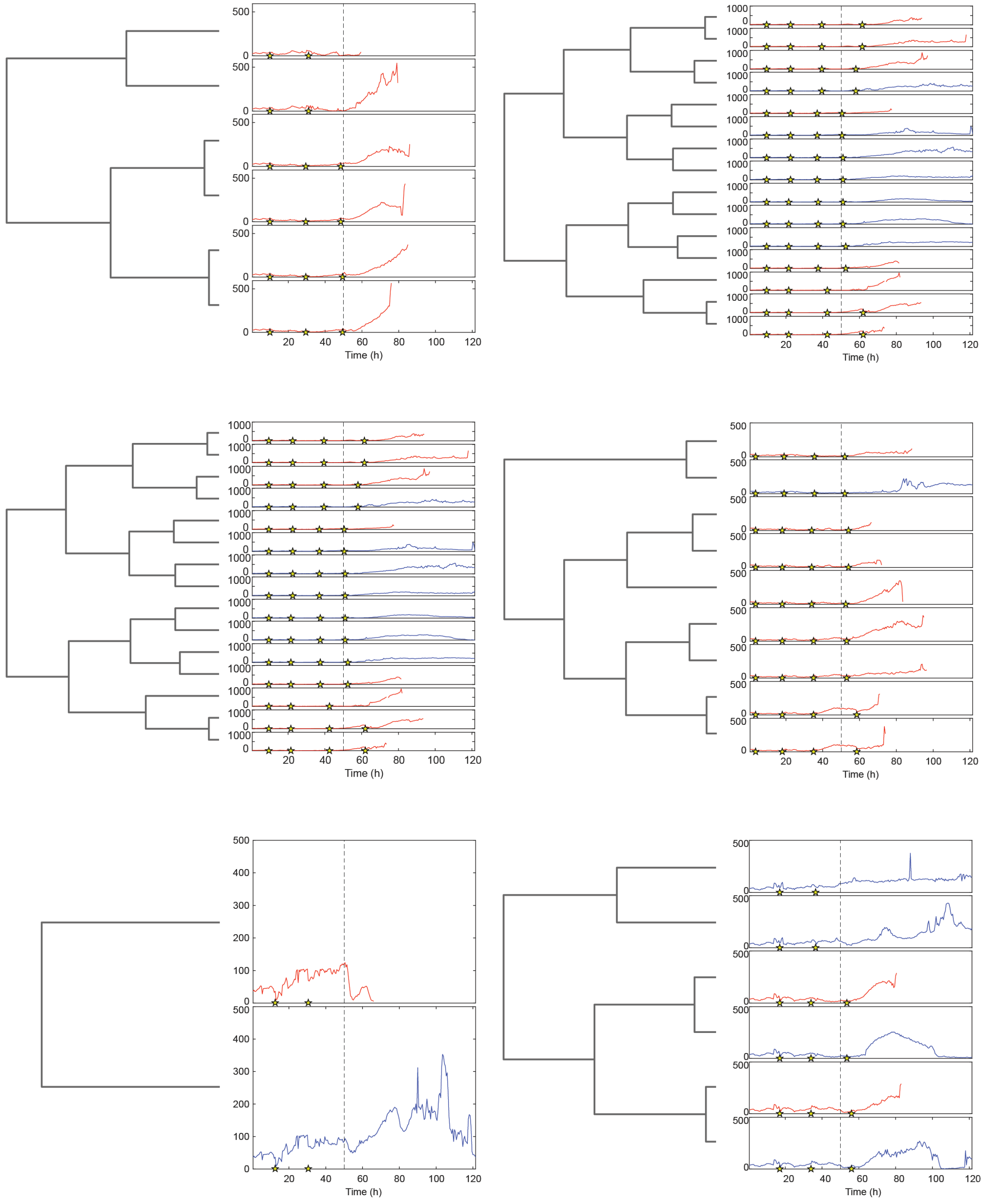

Supplementary Figure 14-2

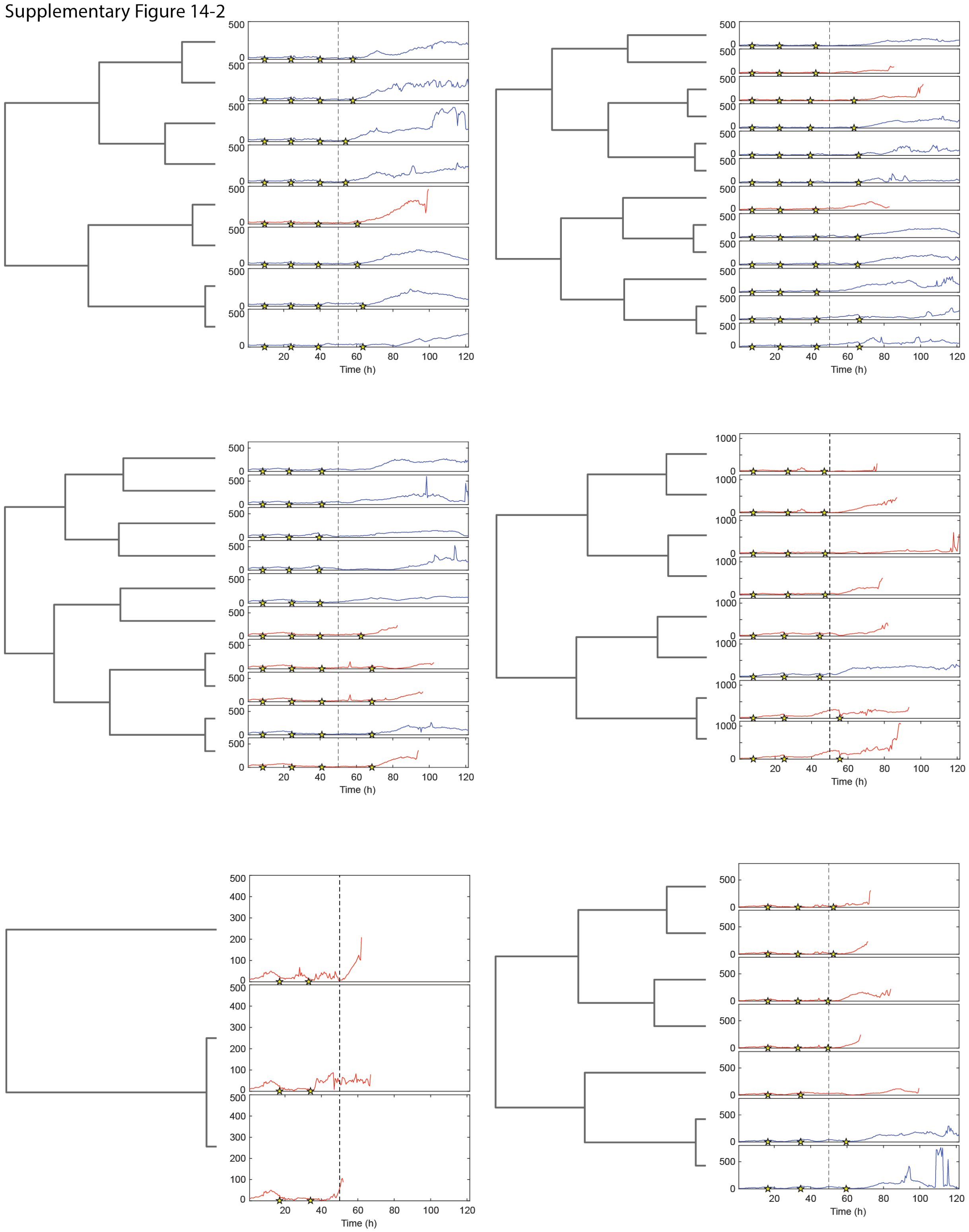

Supplementary Figure 14-3

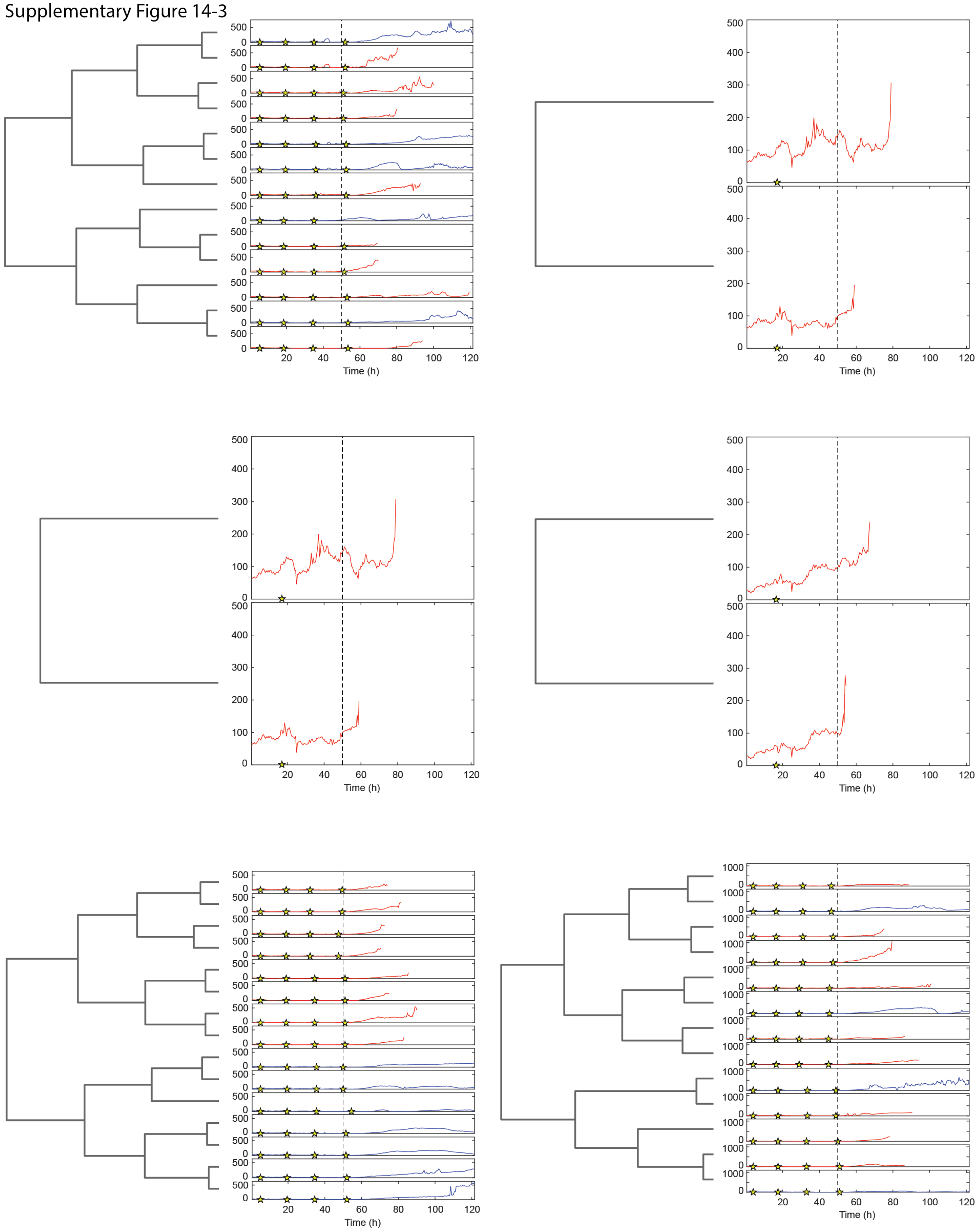

Supplementary Figure 14-4

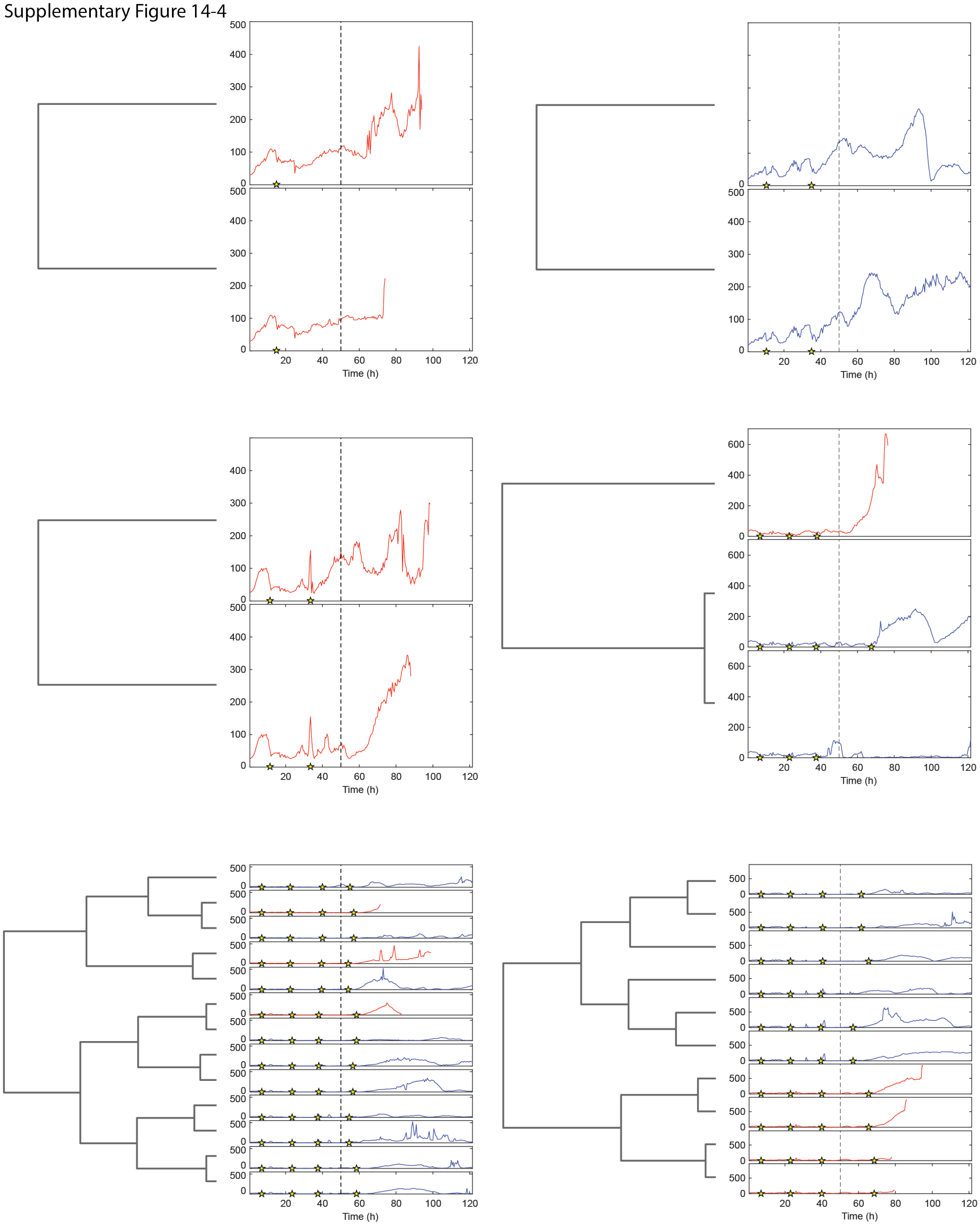

Supplementary Figure 14-5

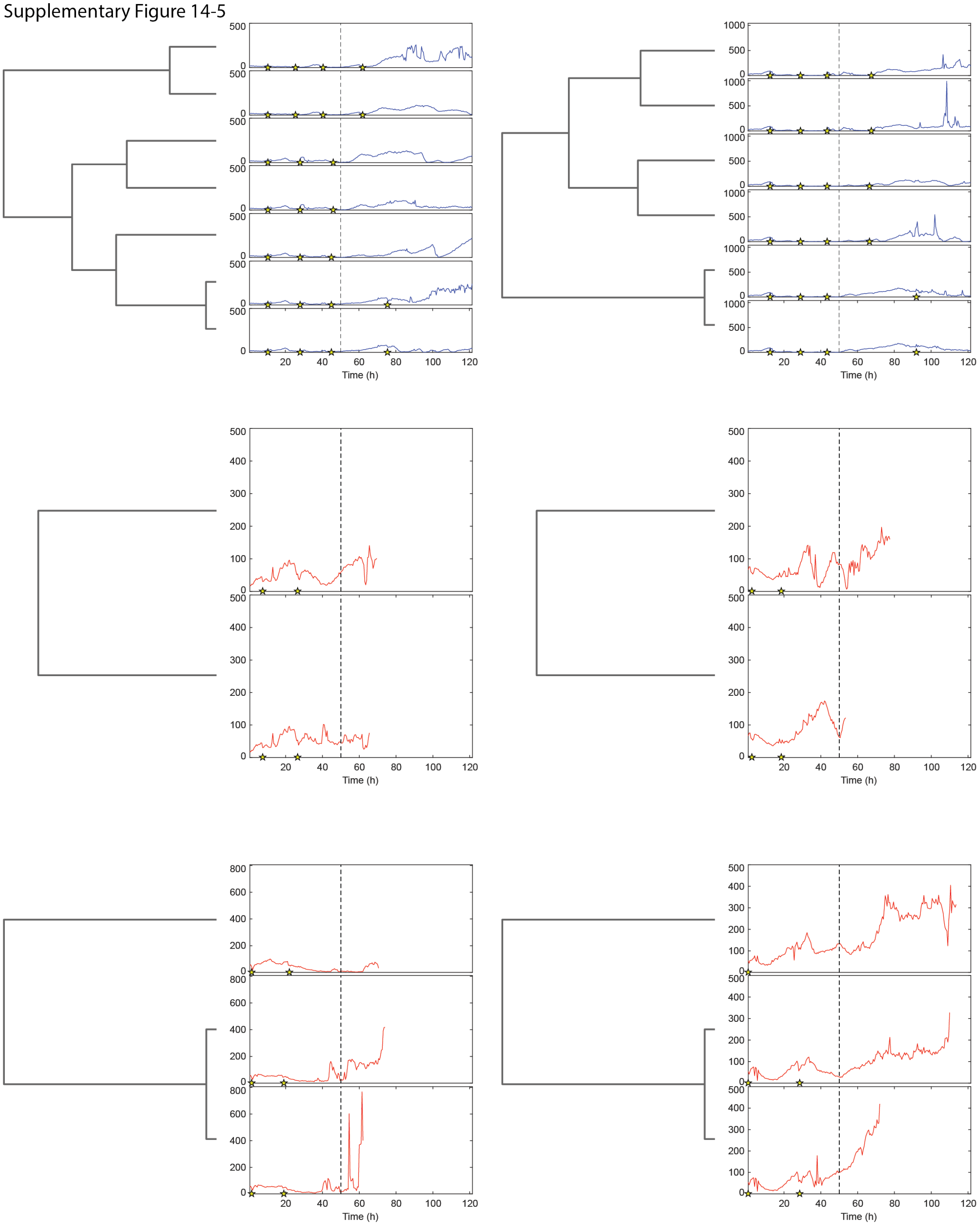

Supplementary Figure 14-6

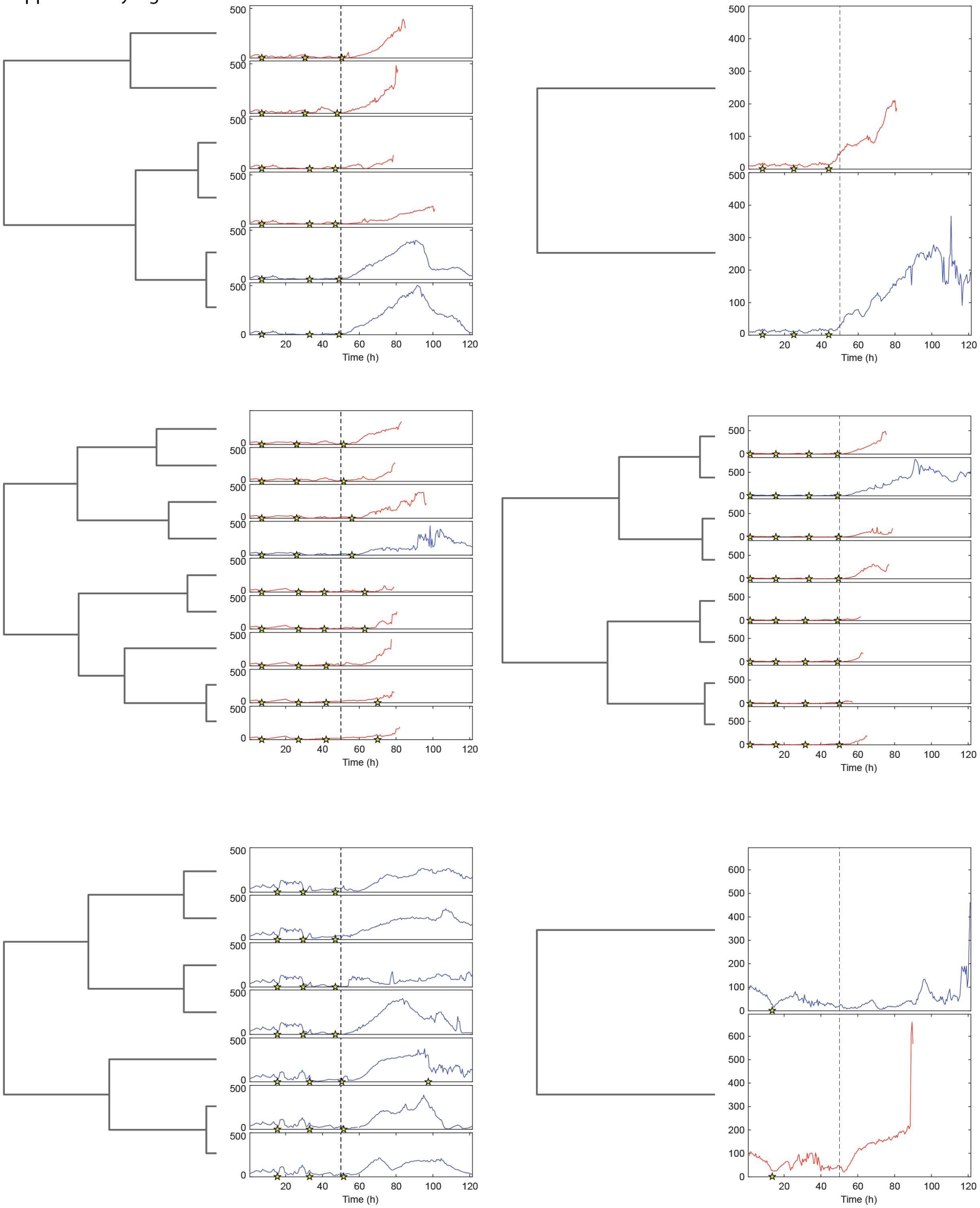

Supplementary Figure 14-7

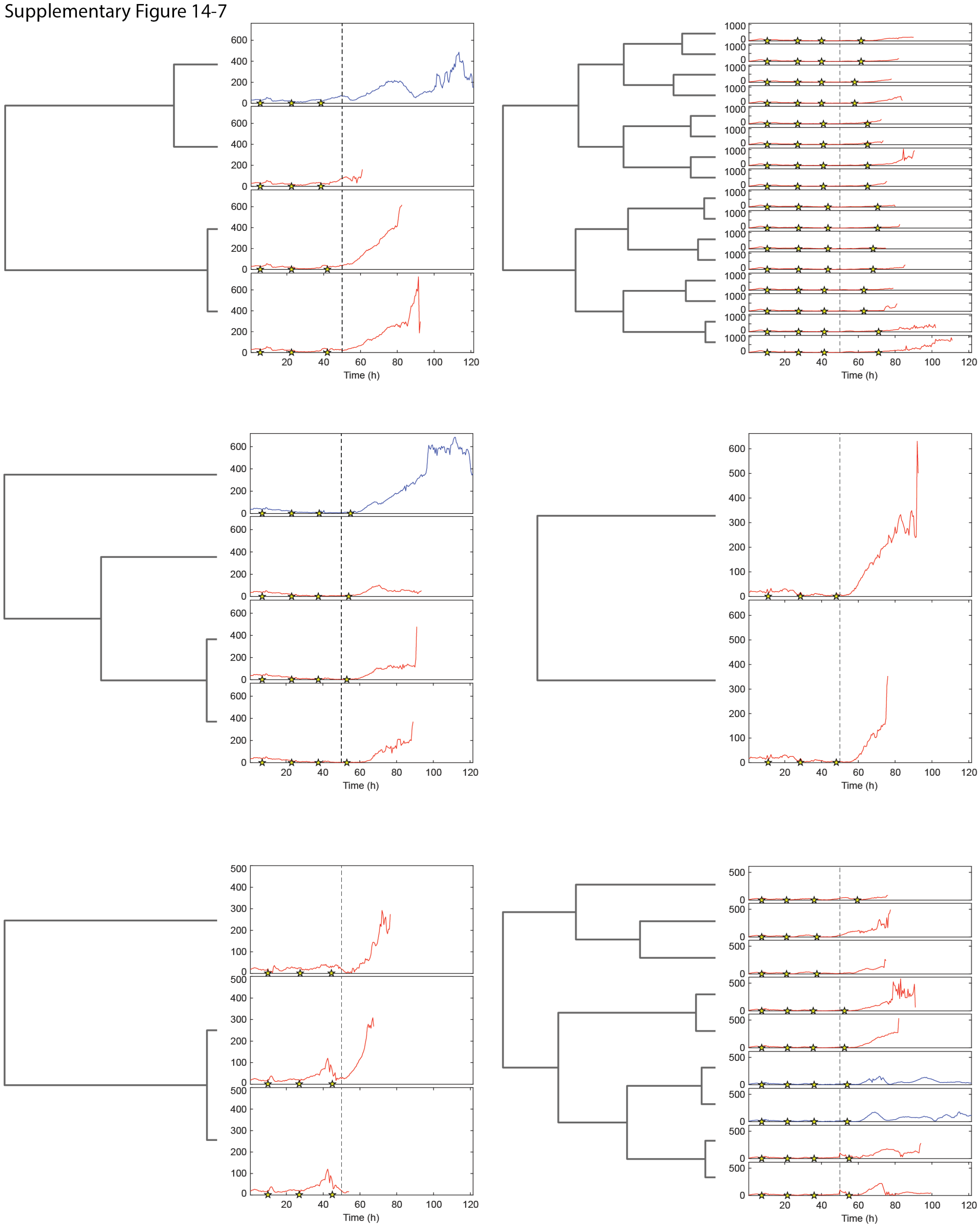

Supplementary Figure 14-8

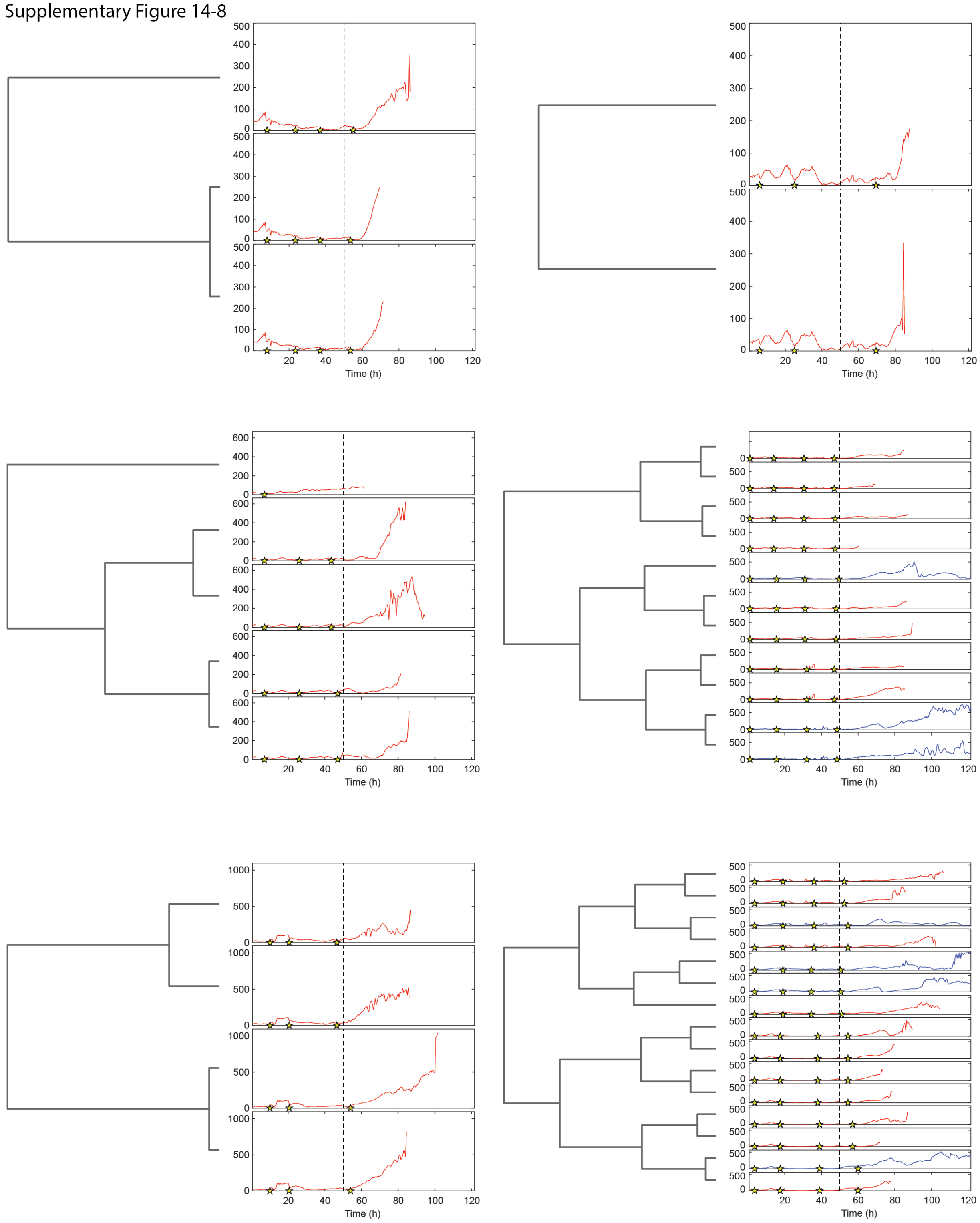

Supplementary Figure 14: *Lineages of cells tracked through the entire experiment.* Plots of cell lineages used for analysis. The cell dendrogram is pictured to the left of each plot, the length of each line corresponds to the time that the cell lasted before it divided, died or the experiment ended. p53-Venus trajectories corresponding to each cell in the dendrogram are plotted to the right. Red traces represent apoptotic cells, blue traces are for surviving cells. Yellow stars represent divisions and the dashed line corresponds to when cisplatin was added.

Supplementary Figure 15-1

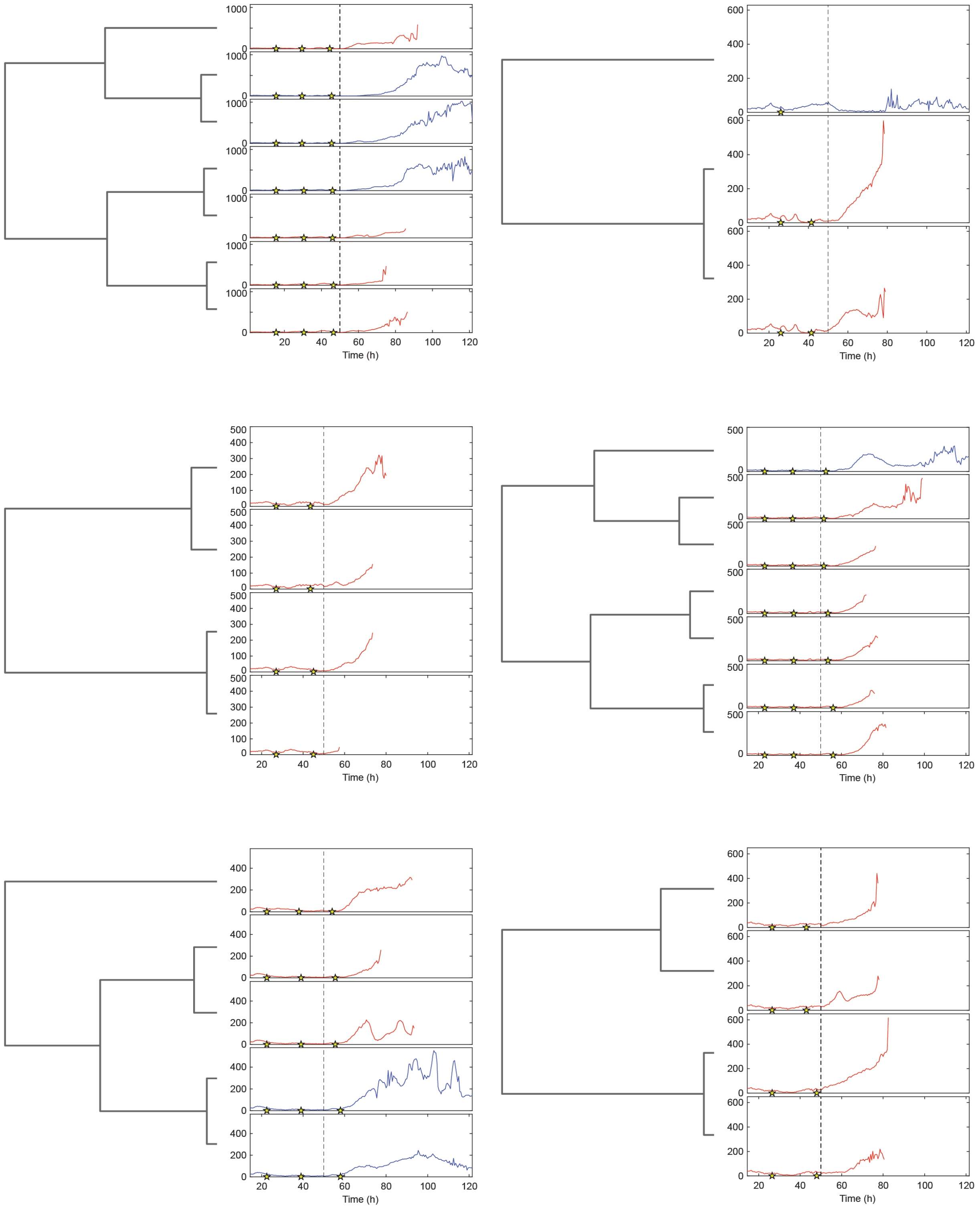

Supplementary Figure 15-2

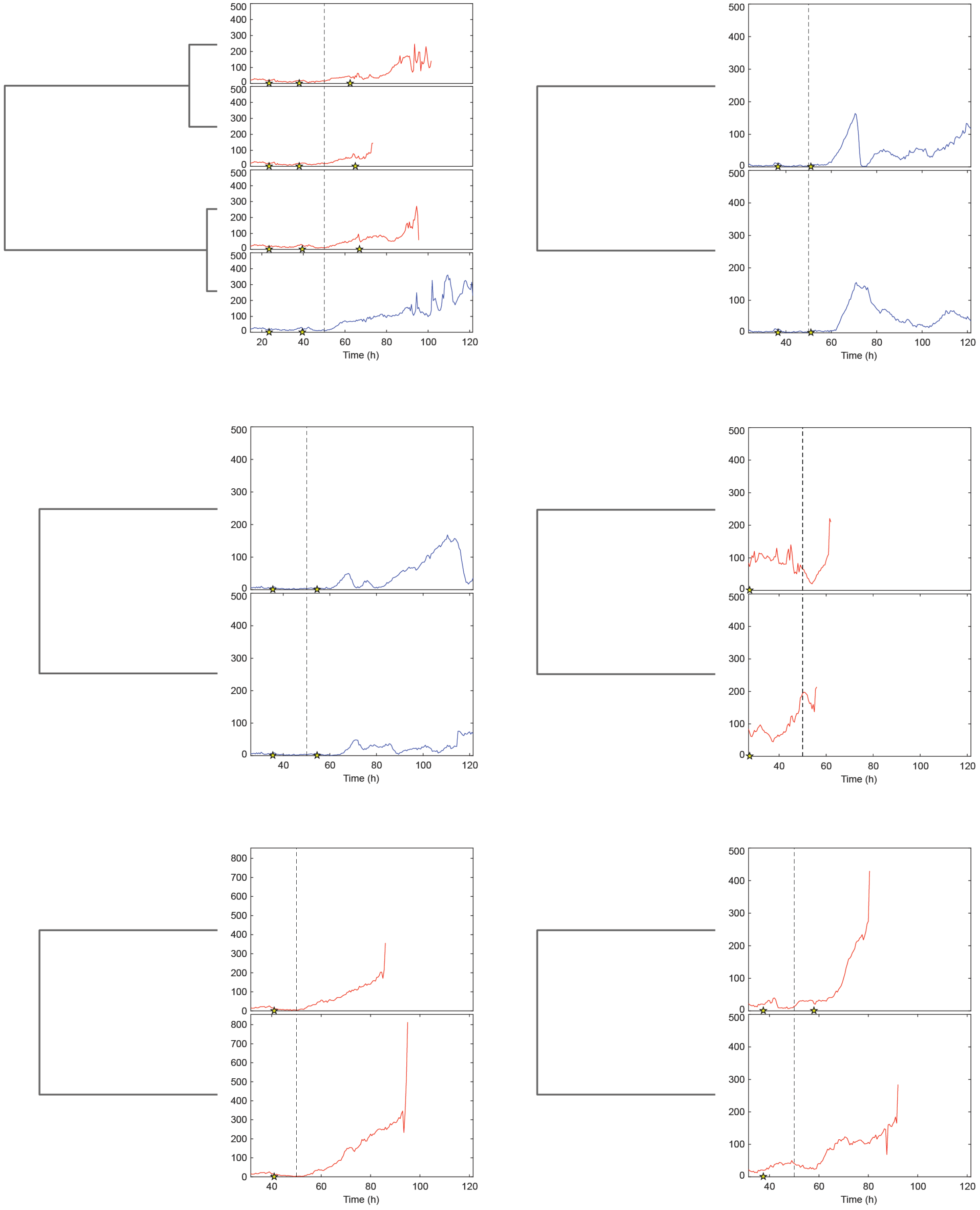

Supplementary Figure 15-3

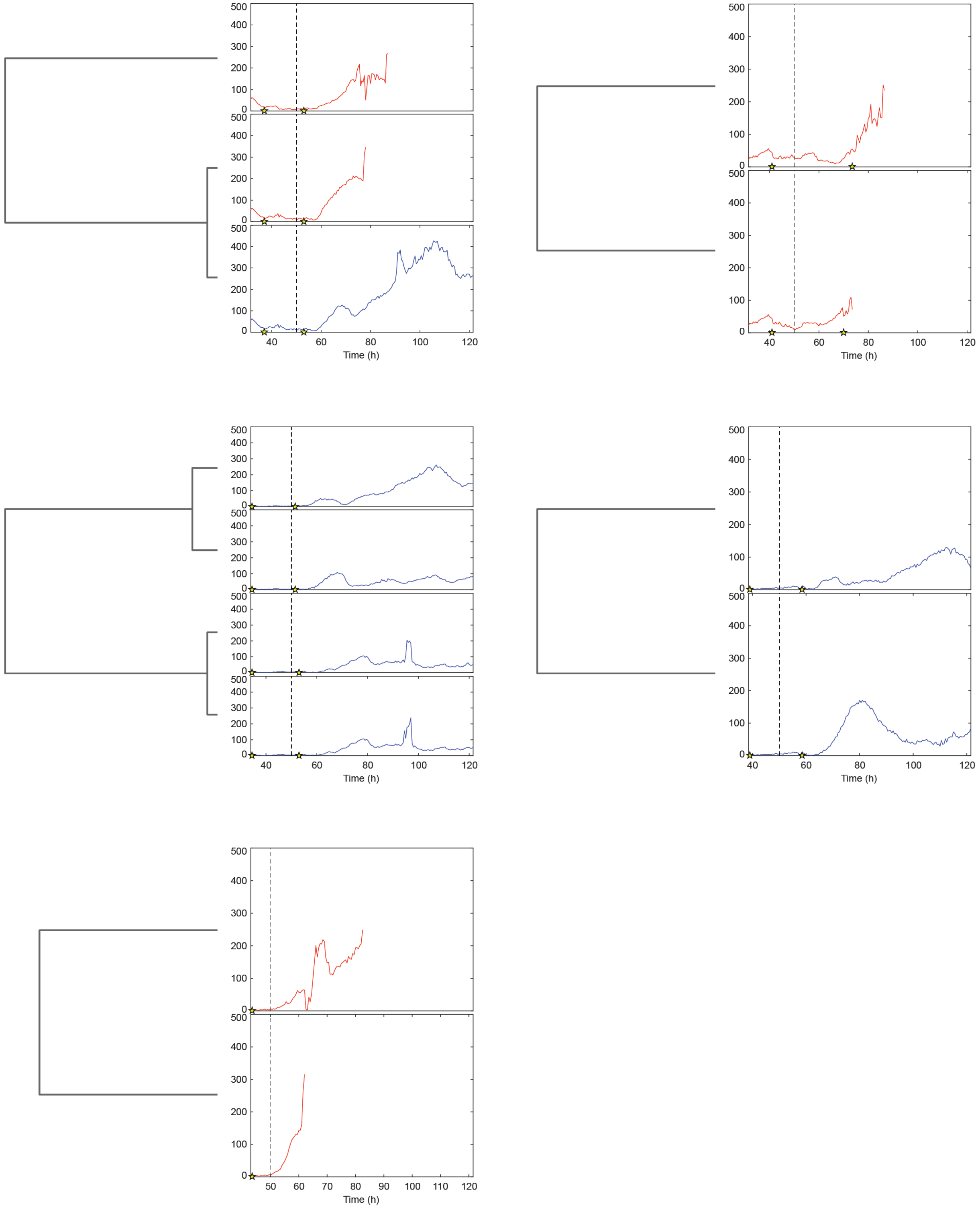

Supplementary Figure 15: *Lineages of cells tracked for a part of experiment.* Plots of cell lineages used for analysis. Since some cells could not be tracked from the beginning to the end of the experiment these cells were tracked starting at a later time point. The cell dendrogram is pictured to the left of each plot, the length of each line corresponds to the time that the cell lasted before it divided, died or the experiment ended. p53-Venus trajectories corresponding to each cell in the dendrogram are plotted to the right. Red traces represent apoptotic cells, blue traces are for surviving cells. Yellow stars represent divisions and the dashed line corresponds to when cisplatin was added.
