# Supplementary material for: Hidden heterogeneity and circadian-controlled cell fate inferred from single cell lineages
Source: Nat Commun. 2018 Dec 18;9:5372. doi: 10.1038/s41467-018-07788-5 (PMC6299096; doi:10.1038/s41467-018-07788-5)
Supplement: Supplementary file 3 — Description of Additional Supplementary Files [file 41467_2018_7788_MOESM3_ESM.docx]

**Description of Supplementary Files**

**File Name:** Supplementary Movie 1

**Description:** Example of a time lapse imaging experiment. The phase, H2B-ECFP and p53-Venus channels are shown. Time stamp is on the bottom left, scale bar on the bottom right. Cisplatin was added 50 hours into the experiment. Three different lineages are tracked and shown in purple, teal and yellow. Tracking was performed using the H2B-ECFP channel and the phase channel was used to keep track of cells for times when the H2B-ECFP channel was ambiguous.

**File Name:** Supplementary Data 1

**Description:** Single cell data. The data is separated into 4 different sheets. In each sheet, the rows correspond to a single tracked cell (394 cells) and the columns correspond to the data at a given timepoint (243 30 minute timepoints). The 4 different sheets are called (i) **Divisions:** This sheet keeps track of all divisions. A ‘1’ indicates that the tracked cell divided at that time. A ‘0’ means the cell did not divide at that time. (ii) **p53:** p53-Venus levels. A ‘-1’ indicates that a cell was not tracked at that timepoint. This could be for two reasons: either the cell died or the cell was not able to be tracked at the beginning of the experiment due to ambiguous identity over time. (iii) **Apoptosis:** A ‘1’ indicates the cell died at that time. (iv) **Lineage:** This sheet contains information on how each cell is related to other cells. If two cells share lineage numbers, then that means they are related. When this changes, i.e. when the cells stop sharing numbers, it means the cell divided.

**File Name:** Supplementary Data 2

**Description:** Detailed explanation of single cell data structure in Supplementary Data 1.
