# Supplementary material for: Hidden heterogeneity and circadian-controlled cell fate inferred from single cell lineages
Source: Nat Commun. 2018 Dec 18;9:5372. doi: 10.1038/s41467-018-07788-5 (PMC6299096; doi:10.1038/s41467-018-07788-5)
Supplement: Supplementary file 6 — Supplementary Data 2 [file 41467_2018_7788_MOESM6_ESM.docx]

**The experiment**:

HCT116 p53-Venus + H2B-ECFP cells were imaged every 30 minutes for 121.5 hours. After 49 hours, or just before frame 98, cells were treated with 12.5 μM cisplatin.

**The data:**

The data is separated into 4 different sheets. In each sheet, the rows correspond to a single tracked cell (394 cells) and the columns corresponds to the data at a given timepoint (243 30 minute timepoints).

**Divisions:** This keeps track of all divisions. A ‘1’ indicates that the tracked cell divided at that time. A ‘0’ means the cell did not divide at that time.

**p53:** p53-Venus levels. A ‘-1’ indicates that a cell was not tracked at that timepoint. This could be for two reasons: either the cell died or the cell could not be tracked at the beginning of the experiment due to ambiguous identity over time.

**Apoptosis:** A ‘1’ indicates the cell died at that time.

**Lineage:** This is how each cell is related. If two cells share lineage numbers, then that means they are related. When this changes, i.e. when the cells stop sharing numbers, that means the cell divided. For example, if we have two cells, cell 1 and cell 2, and the data looks like this:

1 1 1 1 1

1 1 2 2 2

then that means that at the beginning the cell started as a single cell and divided in the third frame. So after frame 3 we have two cells, cell 1 and cell 2, that are related. When there is a ‘0’ that means this cell was not being tracked at this time. Note that if a cell maintains the same number over the entire experiment, that does not indicate that the cell did not divide. The divisions sheet will indicate when the cell divided.
